# IMPDH inhibition enhances cytarabine efficacy in SAMHD1‐expressing leukaemia cells via guanine nucleotide depletion

**DOI:** 10.1002/1878-0261.70226

**Published:** 2026-02-19

**Authors:** Miriam Yagüe‐Capilla, Christopher Dirks, Caroline Eiden, Sonja K. Fesenmayer, Femke M. Hormann, Yolande Klootsema, Ingrid Lilienthal, Si Min Zhang, Nikolas Herold, Sean G. Rudd

**Affiliations:** ^1^ Science for Life Laboratory (SciLifeLab), Department of Oncology‐Pathology Karolinska Institutet Stockholm Sweden; ^2^ Instituto de Biomedicina de Sevilla (IBiS) Hospital Universitario Virgen del Rocío/CSIC/Universidad de Sevilla Spain; ^3^ Departamento de Biología Celular, Facultad de Biología Universidad de Sevilla Spain; ^4^ Childhood Cancer Research Unit, Department of Women's and Children's Health Karolinska Institutet Stockholm Sweden; ^5^ Section of Paediatric Oncology, Astrid Lindgren Children's Hospital Karolinska University Hospital Stockholm Sweden; ^6^ Present address: Internal Medicine V, Hematology, Oncology and Rheumatology, Heidelberg University Hospital Heidelberg Germany

**Keywords:** acute myeloid leukaemia, chemoresistance, dNTP metabolism, drug repurposing

## Abstract

The nucleoside analogue cytarabine (ara‐C) is part of standard treatment against acute myeloid leukaemia (AML). The efficacy of this therapy is dependent upon accumulation of the active triphosphate metabolite ara‐CTP, which mis‐incorporates into genomic DNA, triggering cell death. The deoxyribonucleoside triphosphate triphosphohydrolase (dNTPase) SAMHD1 can hydrolyse ara‐CTP and thereby convert the active metabolite back to its inactive prodrug form. This constitutes a barrier to treatment efficacy and thus strategies to target SAMHD1 are warranted. SAMHD1 activity is allosterically regulated by nucleotides, which are synthesised in cells via distinct pathways. We screened a collection of drugs targeting nucleotide biosynthetic enzymes and identified that inhibition of inosine‐5′‐monophosphate dehydrogenase (IMPDH), responsible for catalysing the rate‐limiting step in guanine nucleotide biosynthesis, sensitises AML cell lines to ara‐C in a SAMHD1‐dependent manner. We show that approved drugs inhibiting IMPDH—mycophenolic acid and ribavirin—imbalance deoxyribonucleoside triphosphate pools and increase ara‐C efficacy in SAMHD1‐proficient, but not deficient, leukaemic cells. Altogether, we provide insight into SAMHD1 regulation in leukaemic cells and show how this process can be exploited by approved drugs to improve ara‐C therapy.

## Graphical abstract

Cytarabine is a key therapy for acute myeloid leukaemia (AML), but its efficacy is limited by the dNTPase SAMHD1, which hydrolyses its active metabolite. Screening nucleotide biosynthesis inhibitors revealed that IMPDH inhibitors selectively sensitise SAMHD1‐proficient AML cells to cytarabine. We propose that IMPDH inhibition disrupts dNTP pools and diminishes SAMHD1 activity, offering a drug‐repurposing strategy to enhance cytarabine response in AML.

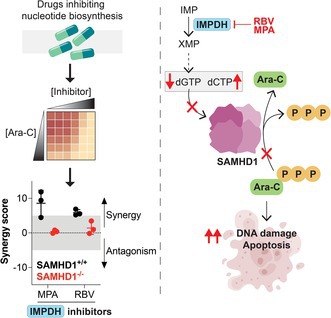

**Abbreviations**
AML: acute myeloid leukaemia
Ara‐C: cytarabine
Ara‐CTP: cytarabine triphosphate
AS1: allosteric site 1
AS2: allosteric site 2
BREQ: brequinar
dGuo: deoxyguanosine
dNTP: deoxyribonucleoside triphosphate
HA: hadacidin
HU: hydroxyurea
IMPDH: inosine‐5′‐monophosphate dehydrogenase
LEF: leflunomide
MPA: mycophenolic acid
PF: pyrazofurin
RBV: ribavirin
RNR: ribonucleotide reductase
SAMHD1: sterile alpha motif and histidine‐aspartic acid domain containing protein‐1

## 1 Introduction

Acute myeloid leukaemia (AML) is characterised by clonal proliferation of myeloid progenitor cells resulting in bone marrow failure and systemic spread of undifferentiated blast cells through the bloodstream. The deoxycytidine analogue cytarabine (ara‐C) is a major component of AML therapy, included in standard induction regimens in combination with the anthracycline daunorubicin, and subsequently used at high doses during consolidation therapy [1]. Outcomes in patients with AML vary considerably and the lack of response to ara‐C is a principal cause of treatment failure and relapse [2]. There is a clear need to exploit our understanding of ara‐C molecular pharmacology and develop strategies to enhance treatment efficacy.

Ara‐C is a nucleoside analogue, a class of chemotherapy prodrugs requiring intracellular bioactivation [3]. Sequential phosphorylation of ara‐C by intracellular nucleoside and nucleotide kinases yields the active triphosphate metabolite ara‐CTP, which is mis‐incorporated into genomic DNA by cellular replicases leading to induction of DNA damage and apoptotic cell death [3]. Sterile alpha motif and histidine‐aspartic acid domain containing protein‐1 (SAMHD1) is a deoxyribonucleoside triphosphate (dNTP) hydrolase that can cleave the triphosphate moiety from ara‐CTP, converting the active cytotoxic metabolite back to its inactive prodrug form [4, 5, 6]. Extensive investigation in AML disease models and patient‐derived material, together with analyses of AML patient cohorts treated with ara‐C [4, 5, 7], all support that SAMHD1 is a major determinant of ara‐C therapy efficacy and highlight this enzyme as an attractive therapeutic target to enhance ara‐C treatments [8].

Efforts to target SAMHD1 have included the use of viral protein‐X (Vpx) as a biologic degrader [4] together with several small molecule screening campaigns and chemical biology approaches yielding various chemotypes capable of SAMHD1 inhibition *in vitro* [6, 9, 10, 11, 12, 13, 14, 15]; however, these approaches are currently restricted to the preclinical setting. To identify cell‐active and potentially clinically actionable pharmacological approaches to target SAMHD1, we previously reported a phenotypic screening campaign that identified existing anticancer drugs, specifically nonallosteric inhibitors of ribonucleotide reductase (RNR), which can be used to indirectly target SAMHD1 [16]. RNR catalyses the rate‐limiting step in *de novo* dNTP biosynthesis, reducing nucleoside diphosphates to their deoxyribonucleoside diphosphate counterparts, and is a long‐standing anticancer target [17, 18]. We established that targeting of RNR with existing cancer therapeutics hydroxyurea (HU) or gemcitabine could sensitise AML models and patient‐derived material to ara‐C therapy in a SAMHD1‐dependent manner. Mechanistically, we proposed that RNR inhibition by HU or gemcitabine causes a dNTP pool imbalance, specifically an inversion of the dATP‐to‐dCTP ratio in leukaemic cells, and that dCTP‐activated SAMHD1 has low ara‐CTPase activity [16]. SAMHD1 dNTP hydrolytic activity is allosterically regulated by nucleotides, with catalytically competent SAMHD1 being a homotetramer requiring nucleotide binding for oligomerisation, specifically binding of guanine nucleotides (GTP/dGTP) at a first allosteric site (allosteric site 1, AS1) and any dNTP occupying a second allosteric site (allosteric site 2, AS2) [19]. This indirect pharmacological approach to target SAMHD1, specifically with the RNR inhibitor HU, is now being evaluated in a Phase I‐II clinical study in adult AML in Sweden [20]. The finding that a particular imbalance in dNTP pool composition can apparently perturb the allosteric regulation of SAMHD1 in leukaemic cells [16] prompted us to ask whether other dNTP pool perturbations can also affect SAMHD1 activity. Such information could inform upon the mechanism of indirect targeting achieved by RNR inhibitors in addition to potentially identifying orthogonal SAMHD1 targeting strategies.

## 2 Materials and methods

### 2.1 Human cell lines and culture

The generation of the CRISPR‐Cas9 engineered derivatives of THP‐1 (ATCC; RRID:CVCL_0006, Manassas, VA, USA), HuT 78 (ATCC; RRID:CVCL_0337) and HL‐60 (ATCC; RRID:CVCL_0002) cell lines has been described previously [4, 16] and these were cultured in Iscove's modified Dulbecco's medium (IMDM, no. 12440061; Thermo Fisher Scientific, Waltham, MA, USA). The cell lines CCRF‐CEM (ATCC; RRID:CVCL_0207), K‐562 (ATCC; RRID:CVCL_0004), MV4‐11 (ATCC; RRID:CVCL_0064), Jurkat (ATCC; RRID:CVCL_0065), MOLT‐4 (ATCC; RRID:CVCL_0013) and MOLT‐16 (DSMZ; RRID:CVCL_1424, Braunschweig, Germany) were cultured in RPMI‐1640 media supplemented with GlutaMAX (no. 61870044; Thermo Fisher Scientific). Culture media was supplemented with 10% heat‐inactivated fetal bovine serum (no. 10500064; Thermo Fisher Scientific) and 50 U·mL^−1^ penicillin, 50 μg·mL^−1^ streptomycin (no. 15070063; Thermo Fisher Scientific). Cells were maintained at 37 °C and 5% CO_2_ in a humidified incubator at densities between 0.1 and 1 million cells·mL^−1^. All cell lines were authenticated by Microsynth AG (Balgach, Switzerland) through STR genotyping (markers used were D3S1358, TH01, D21S11, D18S51, PentaE, D5S818, D13S317, D75820, D16S539, CSF1PO, PentaD, AMEL, vWA, D8S1179, TPOX and FGA) and the absence of mycoplasma was routinely confirmed using the MycoAlert Mycoplasma Detection Kit (no. LT07‐318; Lonza, Basel, Switzerland).

### 2.2 Compound preparation

Compounds were dissolved in dimethyl sulfoxide (DMSO) at 10–50 mm and stored at −20 °C. Hydroxyurea (HU) was prepared immediately prior to the experiment. Cytarabine (ara‐C, no. C1768), hydroxyurea (HU, no. H8627), mycophenolic acid (MPA, no. M3536), brequinar (BREQ, no. SML0113), leflunomide (LEF, no. L5025), pyrazofurin (PF, no. SML1502) and deoxyguanosine (dGuo, no. D0901) were purchased from Sigma Aldrich (St. Louis, MO, USA). Ribavirin (RBV, no. OT‐0885) was obtained from Combi‐Blocks (San Diego, CA, USA); hadacidin (HA, no. sc‐490 177) from Santa Cruz Biotechnology (Dallas, TX, USA). Lomofungin (NSC106995) was provided by the National Cancer Institute (NCI)/Division of Cancer Treatment and Diagnosis (DCTD)/Developmental Therapeutics Program (DTP) (Rockville, MD, USA).

### 2.3 Proliferation inhibition assay and synergy measurements

Proliferation inhibition assays and synergy measurements were performed as previously described [16, 21]. Compounds dissolved in DMSO were dispensed using the Tecan D300e Digital Dispenser in black 384‐well plates with clear flat bottom (no. CLS3764; Corning, Corning, NY, USA) and DMSO normalised across the plate, not exceeding 1% volume per well. Cells (2000 per well) were seeded using a MultiDrop Combi Reagent Dispenser (Thermo Fisher Scientific) in a final 50 μL volume of their corresponding media. The plates were incubated in a humidified chamber for 96 h before addition of resazurin (Sigma Aldrich; no. R7017) at a final 0.01 mg·mL^−1^ concentration. After 6 h, fluorescence intensity was measured using a Hidex Sense Microplate Reader at 544/590 nm excitation/emission wavelengths. Relative cell proliferation was calculated using the fluorescence from positive control wells (corresponding to cells and normalised DMSO) as 100% viability and those from negative control wells (containing only normalised DMSO diluted in media) as 0% viability. graphpad prism v10.2.0 software was used to fit the resulting curves to a nonlinear regression model (variable slope, four parameter) and obtain a matrix with the average percentage of cell viability from the technical duplicates, which was used as input in the R‐package Synergyfinder v2.0 [22] or v3.0 [23] to calculate the average delta score (ZIP, Bliss, HSA) across the drug concentration matrix. According to Yadav *et al*. [24] synergy scores higher than 5 corresponded to synergism, values between −5 and 5 were noted as additive interaction, and delta scores lower than −5 were considered as strong antagonism.

### 2.4 Immunoblots

Protein lysates were collected from 1‐ to 5‐million cells per sample. Cell pellets were washed twice in PBS and resuspended in lysis buffer (50 mm Tris HCl pH 8, 150 mm NaCl, 1 mm EDTA, 1% NP‐40, 0.1% SDS) containing cOmplete EDTA‐free protease inhibitor cocktail (no. 04693159001; Roche, Basel, Switzerland) and Halt phosphatase inhibitor cocktail (no. 78426; Thermo Fisher Scientific) before incubation on ice for 45–60 min and frequent vortexing. Cellular debris was removed from the lysates by full‐speed centrifugation for 20 min. To normalise the protein content loaded per well, the protein concentration was first quantified using the Pierce BCA Protein Assay Kit (no. 23225; Thermo Fisher Scientific), following the instructions indicated by the manufacturer, and using a Hidex Sense Microplate Reader to determine absorbance at 582 nm. Samples of total 10–20 μg protein were prepared in NuPAGE LDS Sample Buffer 4× (no. NP0007; Thermo Fisher Scientific) and NuPAGE Sample Reducing Agent 10X (no. NP0004; Thermo Fisher Scientific) and denatured at 95 °C for 7 min. Sample lysates and protein standards (Precision Plus Protein Dual Color Standards, no. 1610374; Bio‐Rad, Hercules, CA, USA) were loaded into Criterion TGX 4–20% precast polyacrylamide gels (Bio‐Rad) for sodium dodecyl sulfate‐polyacrylamide gel electrophoresis (SDS/PAGE) and further transfer to a nitrocellulose membrane using the Trans‐Blot Turbo Transfer System (Bio‐Rad). Blocking was performed at room temperature (RT) in TBS‐T (Tris‐buffered saline, 0.1% Tween 20) + 5% milk for 1 h and membranes were incubated with primary antibodies overnight at 4 °C. After three subsequent TBS‐T washes, 1 : 10 000 IRDye conjugated (no. 926‐32213, 926‐32212, 926‐68073, 926‐68072; Li‐Cor, Lincoln, NE, USA) secondary antibodies were probed (45 min at RT) and target proteins were visualised on an Odyssey Fc Imaging System (Li‐Cor) and analysed using Image Studio Lite v5.2 (Li‐Cor). When chemiluminescent was measured, 1 : 5000 HRP‐conjugated secondary antibodies (Peroxidase AffiniPure Donkey Anti‐Rabbit IgG (H + L), no. 711–035‐152; Jackson Immunoresearch, West Grove, PA, United States) were used in combination with 1 : 1 of the SuperSignal™ West Pico PLUS Luminol/Enhancer Solution and SuperSignal™ West Pico PLUS Stable Peroxide Solution (SuperSignal™ West Pico PLUS Chemiluminescent Substrate Kit, no. 34580; Thermo Fisher Scientific). Densitometry analyses were performed using imagestudio Software (version 6.0; Li‐Cor) and values normalised to a loading control. The reference and dilutions of the primary antibodies used in this manuscript are the following: SAMHD1 (72.2 kDa, 1 : 1000, no. ab128107; Abcam (Cambridge, UK), or 1 : 4000 no. A303‐691A; Bethyl), IMPDH (55.4 kDa, 1 : 500, no. sc166551; Santa Cruz Biotechnology), SOD‐1 (15.9 kDa, 1 : 500, no. sc‐17767; Santa Cruz Biotechnology), cleaved‐PARP (89 kDa, 1 : 500, no. 9541S; Cell Signaling, Danvers, MA, USA), γH2AX (15 kDa, 1 : 1000, no. 2577S; Cell Signaling), β‐actin (42 kDa, 1 : 4000, no. ab6276; Abcam).

### 2.5 Enzyme‐coupled SAMHD1 activity assay

The enzyme‐coupled SAMHD1 activity assay was performed as detailed previously [25]. Briefly, recombinant proteins used in the assay (human SAMHD1 and *Escherichia coli* inorganic pyrophosphatase, or PPase) were expressed and purified as described [15, 20] and kept until further use at −80 °C in storage buffer (20 mm HEPES pH 7.5, 300 mm NaCl, 10% glycerol, 2 mm TCEP buffer). Compounds were twofold serially diluted in 96‐well clear V‐bottomed polystyrene microplates (no. CLS3896; Corning). The enzymatic reaction was performed in 384‐well clear microplates (cat no. CLS3701; Corning), first adding the diluted drug to be tested, the enzymes (0.35 mm SAMHD1 and 12.5 U·mL^−1^ PPase) and the SAMHD1 allosteric regulator and substrate dGTP (25 μm, no. 27‐1870‐04; GE Healthcare, Chicago, IL, USA) diluted in reaction buffer (25 mm Tris‐acetate pH 8, 40 mm NaCl, 1 mm MgCl_2_, 0.005% Tween‐20, 0.3 mm TCEP). After 20 min at RT, the reaction was quenched by 7.9 mm EDTA. Subsequently, it was incubated with malachite green reagent (0.5 mm malachite green, 0.28% ammonium molybdate, 0.036% Tween‐20) for 20 min at RT. Absorption at 630 nm was measured with a Hidex Sense microplate reader to monitor the total inorganic phosphate released in the reaction. graphpad prism v10.2.0 software was used to normalise the absorbance values to the positive (reaction with SAMHD1, PPase and dGTP; 100% SAMHD1 activity) and negative (dGTP alone; 0% SAMHD1 activity) controls to obtain the relative SAMHD1 activity.

### 2.6 Cellular thermal shift assay (CETSA)

THP‐1 cells at densities of 1 or 0.5 million cells·mL^−1^ were incubated with the compounds to be evaluated for 3 or 24 h, respectively, prior to collection of 12 million cells per condition. After PBS wash, cell samples were resuspended in PBS containing cOmplete EDTA‐free protease inhibitor cocktail (no. 04693159001; Roche) and aliquoted at 1 million cells per PCR tube. Samples were then heated for 3 min at temperatures ranging from 34.5 to 62 °C. After resting for 5 min at RT, cells were resuspended in lysis buffer (250 mm HEPES pH7.5, 25 mm β‐glycerophosphate, 0.5 mm Na_3_VO_4_, 50 mm MgCl_2_, 10 mm TCEP, 1X cOmplete, Mini EDTA‐free protease inhibitor cocktail), followed by three freeze‐thawing cycles (5:5 min of ethanol‐dry ice and 37 °C bath each cycle) and cold centrifugation at 20 000× ***g*** for 20 min to remove aggregated proteins. Lysates were transferred to a new tube and for preparation for immunoblotting. Protein band intensities were quantified and normalised to SOD‐1 as thermostable protein loading control [26] before curve fitting using Boltzmann sigmoidal model in graphpad prism v10.2.0 software.

### 2.7 dNTP measurement by coupled click chemistry and DNA polymerase‐based assay

Cells were cultured at 0.5 million cells·mL^−1^ for treatment. Each 24 h, 5 million cells per condition were PBS‐washed, harvested and flash frozen in a dry ice‐ethanol bath for further storage at −80 °C. To extract the dNTPs, cell pellets were resuspended in a cold 1 : 1 (v : v) methanol/water solution and were subjected to two alternating cycles of 10 min in dry ice‐ethanol bath and ice, followed by cold centrifugation at 16 000× ***g*** for 20 min. The supernatant was transferred to a clean tube and the procedure was repeated. Each sample was aliquoted in four tubes corresponding to 1 million cells and the supernatants were dried under vacuum. The four canonical dNTPs (dTTP, dCTP, dATP and dGTP) were quantified using a modified DNA polymerase assay coupled to click reaction, as described [27]. Dried supernatants were dissolved in 100 μL DNA polymerase reaction buffer (for dATP, dCTP and dGTP determination: 0.04 U·μL^−1^ Illustra rTaq DNA Polymerase, 1X Taq DNA Polymerase Buffer, 0.6 μm oligonucleotides, 40 μm 5‐ethynyl‐dUTP; for dTTP quantification: 0.01 U·μL^−1^ Vent(exo‐) DNA Polymerase, 1X Vent Reaction Buffer, 0.6 μm oligonucleotides, 5 μm C8‐Alkyne‐dCTP) and incubated at 60 °C for 40 min. Samples were transferred to a 96‐well clear bottom black microplate (0.5 million cells, 50 μL reaction per well in technical duplicates) containing 50 μL of streptavidin sepharose beads (no. 17‐5113‐01; GE Healthcare), and were incubated for 30 min at RT in agitation for pull‐down. After DNA denature with NaOH 0.1 m for 5 min, beads were washed twice with wash buffer (10 mm Tris–HCl pH 7.5, 1 mm EDTA and 0.1% SDS) and four times with di‐distilled water. Conjugation of the TAMRA fluorophore to the incorporated 5‐ethynyl‐dUTP/C8‐Alkyne‐dCTP was performed through copper(I)‐catalysed alkyne‐azide cycloaddition (CuAAC) or click reaction. Thus, samples were incubated with 100 μL click reaction buffer (10 μm TAMRA‐Azide, 0.5 mm CuSO_4_, 2.5 mm THPTA, 5 mm sodium ascorbate) for 1 h in agitation protected from light. Following washing steps, fluorescence was detected using Tecan Spark 10m at 529/575 nm excitation/emission wavelengths.

### 2.8 Annexin V apoptosis assay

Following drug treatment, cells were then harvested by centrifugation at 400× ***g*** for 5 min, washed twice by PBS and then stained for BD Pharmingen™ Alexa Fluor™ 647 Annexin V (567356; BD Biosciences, Franklin Lakes, NJ, USA) and PI (51‐66211E; BD Biosciences) in 1X binding buffer (51‐66121E; BD Biosciences) per manufacturer's protocol. Samples were subsequently acquired at a minimum of 10 000 events per sample on an Attune NxT Flow Cytometer (Thermo Fisher Scientific), which is further analysed using flowjo. Specifically, cells were first gated out based on forward (FSC) and side (SCC) scattering, followed by doublet discrimination based on the FSC height and area. Viable, early apoptotic, apoptotic and necrotic cells were then identified via quadrant gating on the single cell population, based on Annexin‐V‐Alexa 647 and PI signals.

### 2.9 Statistical analysis

Statistical analyses were performed in graphpad prism v10.2.0 software and *P* values are represented as asterisks in the figures (*P < 0.05, **P < 0.01 and ***P < 0.001). The specific statistical method used and number of experimental and technical replicates are specified in the figure legends.

## 3 Results

### 3.1 A focused drug‐combination screen identifies inhibitors of IMPDH as cytarabine‐sensitisers in SAMHD1‐proficient leukaemia cells

Through an unbiased phenotypic screening approach, we previously identified that nonallosteric inhibitors of RNR can sensitise leukaemic cells to ara‐C in a SAMHD1‐dependent manner, thus establishing an indirect pharmacological approach to target SAMHD1. Mechanistically, we proposed a model in which the changes in dNTP pools caused by RNR inhibition perturb the allosteric activation of SAMHD1 at allosteric site 2 (AS2), which alters substrate specificity, specifically that dCTP‐activated SAMHD1 is a poor ara‐CTPase [16]. Here, we wanted to investigate this phenomenon further and ask whether targeting nucleotide biosynthetic enzymes upstream of RNR, thus inducing distinct dNTP pool imbalances, could also yield SAMHD1‐dependent sensitisation to ara‐C. We reasoned this could offer insight into the molecular mechanisms underpinning indirect pharmacological targeting of SAMHD1 in addition to potentially uncovering orthogonal SAMHD1‐targeting strategies.

**Fig. 1:**
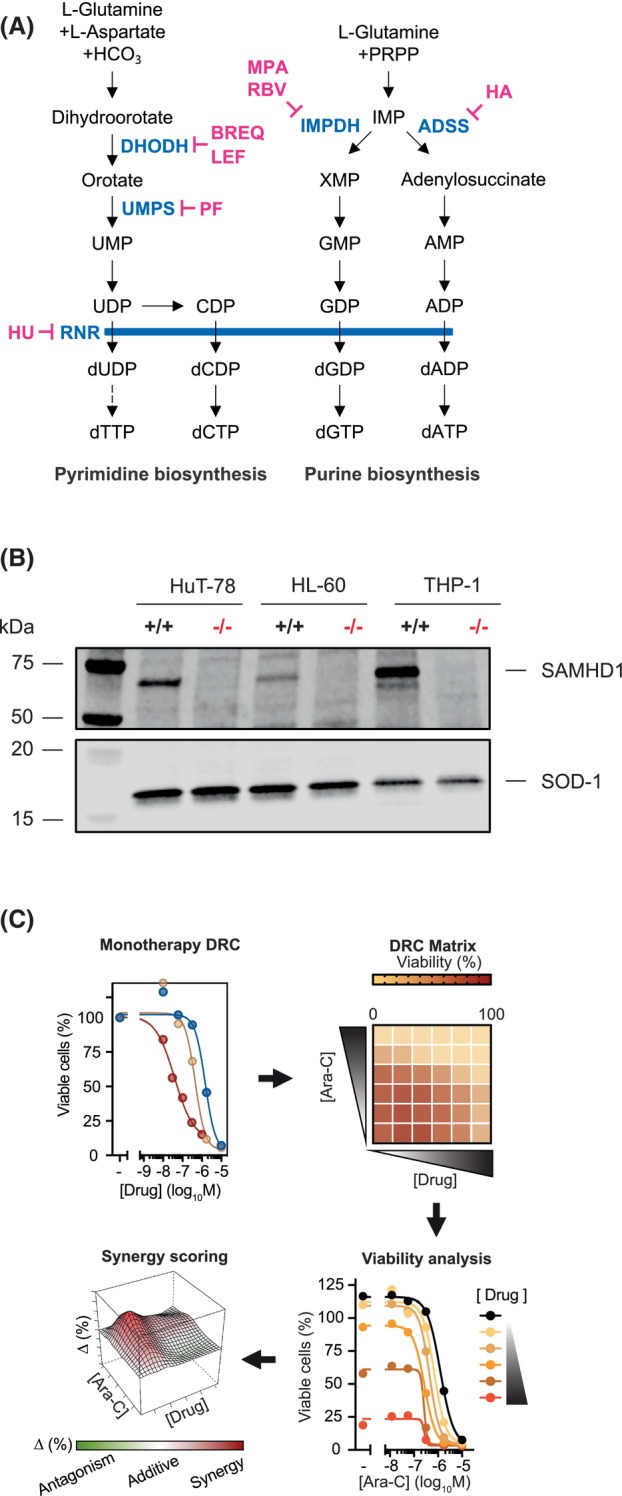
Overview of drug‐combination screen of nucleotide biosynthesis inhibitors and cytarabine in SAMHD1‐proficient and deficient cells. (A) *De novo* synthesis of pyrimidine (left) and purine (right) deoxyribonucleoside triphosphates (dNTPs). Depicted in the pathway are the compounds selected for the screening (pink) and the respective enzymes targeted by them (blue). HA (hadacidin), BREQ (brequinar), LEF (leflunomide), PF (pyrazofurin), HU (hydroxyurea), DHODH (dihydroorotate dehydrogenase), UMPS (uridine monophosphate synthetase), RNR (ribonucleotide reductase), RBV (ribavirin), MPA (mycophenolic acid), IMPDH (inosine‐5′‐monophosphate dehydrogenase) and ADSS (adenylosuccinate synthetase). (B) Immunoblot analysis of lysates prepared from SAMHD1‐proficient and deficient cell line panel. Representative image shown (*n* = 2). (C) Schematic of screening pipeline. Briefly, monotherapy dose response curves (DRC) of the selected nucleotide biosynthesis inhibitors were first performed to determine the optimal concentration ranges, after which cells were subjected to a DRC matrix comprised of cytarabine (ara‐C) and the nucleotide biosynthesis inhibitor. Output of the screen was cell viability measurements and drug‐combination synergy scores.

To this end, we compiled a collection of compounds inhibiting distinct steps of *de novo* purine and pyrimidine biosynthesis (Fig. 1A) and tested them in several SAMHD1‐proficient and deficient cell line pairs (Fig. 1B) for their ability to elicit SAMHD1‐dependent synergy with ara‐C in a proliferation inhibition assay. We devised a workflow [21] (Fig. 1C) which began by determining the concentration range for each compound to produce full monotherapy dose–response curves. Next, with this concentration range, the cell line pairs were exposed to concentration matrices of ara‐C in combination with the nucleotide biosynthesis inhibitors and from the resulting proliferation inhibition data, drug‐combination scores were calculated using several synergy models, including the zero‐interaction potency (ZIP) model [24]. Drug‐combination summary scores were then derived from the dose–response landscapes and compared. We included hydroxyurea (HU), an inhibitor of RNR, as a positive control in our screen [16].

**Fig. 2:**
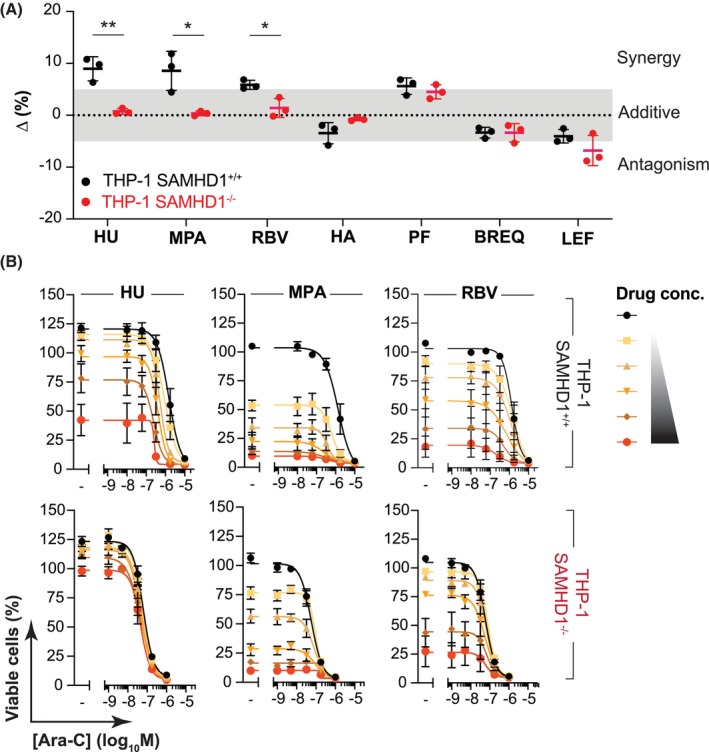
Focused drug‐combination screen identifies inhibitors of IMPDH as cytarabine‐sensitisers in SAMHD1‐proficient leukaemia cells. (A) Drug synergy plots for cytarabine (ara‐C) and the indicated nucleotide biosynthesis inhibitor in *SAMHD1* ^+/+^ or *SAMHD1* ^−/−^ THP‐1 cells. Each data point indicates an average delta score based on the ZIP model from a single dose–response matrix experiment performed in duplicate. Shaded area depicts delta score from −5 to 5, which corresponds to an additive effect. *N* = 3, mean ± SD represented with horizontal and vertical bars, respectively. Two‐tailed unpaired *t*‐test: *P < 0.05 and **P < 0.01. HU (hydroxyurea), MPA (mycophenolic acid), RBV (ribavirin), HA (hadacidin), PF (pyrazofurin), BREQ (brequinar), LEF (leflunomide). (B) Proliferation inhibition analysis of ara‐C and IMPDH inhibitor, or HU, combination treatment in *SAMHD1* ^+/+^ or ^−/−^ THP‐1 cells. Mean values from three to four independent experiments (*n* = 3 *SAMHD1* ^+/+^ cells, *n* = 4 for *SAMHD1* ^−/−^ cells) each performed in duplicate are plotted; error bars indicate SEM. Drugs: HU, 10–100 μm; MPA, 0.5–5 μm; RBV, 5–150 μm; ara‐C, 0.01–10 μm for *SAMHD1* ^+/+^ cells and 0.001–1 μm for *SAMHD1* ^−/−^ cells.

Data derived from the THP‐1 cell line pair are shown in Fig. 2A and Figs S1, S2 and the HuT 78 and HL‐60 cell line pairs are shown in Figs S3, S4. The positive control, HU, consistently resulted in synergy when combined with ara‐C in SAMHD1‐proficient models that was significantly reduced in the SAMHD1‐deficient counterparts to a near additive effect (Fig. 2B and Figs S1, S3), consistent with our previous findings [16]. Out of the compound collection, only mycophenolic acid (MPA) and ribavirin, two chemically distinct inhibitors of inosine‐5′‐monophosphate dehydrogenase (IMPDH) [28], elicited a SAMHD1‐dependent drug‐combination effect with ara‐C (Fig. 2B and Figs S1, S3). MPA is the active metabolite of mycophenolate mofetil (MMF), an FDA‐approved immunosuppressant used during solid organ transplantation and certain haematopoietic stem cell transplantation protocols, whilst ribavirin is an FDA‐approved broad‐spectrum antiviral. The SAMHD1‐dependent ara‐C sensitisation by MPA and ribavirin was most prominent in the THP‐1 cell line pair, in which synergy was observed in the SAMHD1‐proficient cell line but a significant reduction to an additive response was observed in the SAMHD1‐deficient line (Figs 2B and S1). In SAMHD1‐proficient HuT 78 cells, MPA and ribavirin combined in an additive manner with ara‐C, but this drug–drug interaction was reduced in the absence of SAMHD1 (Figs S3, S4), whilst in HL‐60 cells, which express comparatively little SAMHD1 (Fig. 1B), they combined in a weakly antagonistic manner and no effect of SAMHD1 status was observed (Figs S3, S4). IMPDH is responsible for the conversion of the purine precursor inosine monophosphate (IMP) to xanthosine monophosphate (XMP), which is the rate‐limiting step in guanine nucleotide biosynthesis, but IMP is also a substrate for adenylosuccinate synthetase (ADSS) required to produce adenosine nucleotides. However, targeting ADSS with hadacidin [29] yielded largely additive effects in combination with ara‐C and did not consistently differ between SAMHD1 proficient and deficient cells (Fig. 2B and Figs S1, S3). Finally, targeting of pyrimidine nucleotide biosynthesis, either via DHODH inhibition with brequinar or leflunomide, or UMP synthase inhibition with pyrazofurin [30], yielded no SAMHD1‐dependent synergy with ara‐C (Fig. 2B and Figs S1, S3). Taken together, our focused drug‐combination screen identified ribavirin and MPA, both approved drugs and chemically distinct inhibitors of IMPDH, as being an additional class of compounds capable of eliciting SAMHD1‐dependent synergy with ara‐C.

### 3.2 IMPDH inhibitors MPA and ribavirin do not directly inhibit SAMHD1 dNTPase activity

Next, we sought to investigate the mechanism underpinning the SAMHD1‐dependent drug–drug interaction between ara‐C and IMPDH inhibitors ribavirin and MPA. IMPDH catalyses the rate‐limiting step in guanine nucleotide biosynthesis, and guanine nucleoside triphosphates (GTP and dGTP) are essential for the allosteric activation of SAMHD1 dNTPase activity via AS1 and AS2. Thus, we hypothesised that inhibition of IMPDH will deplete guanine nucleotide pools and disrupt SAMHD1 activation, which could provide an orthogonal indirect strategy to perturb SAMHD1 activity in cancer cells.

**Fig. 3:**
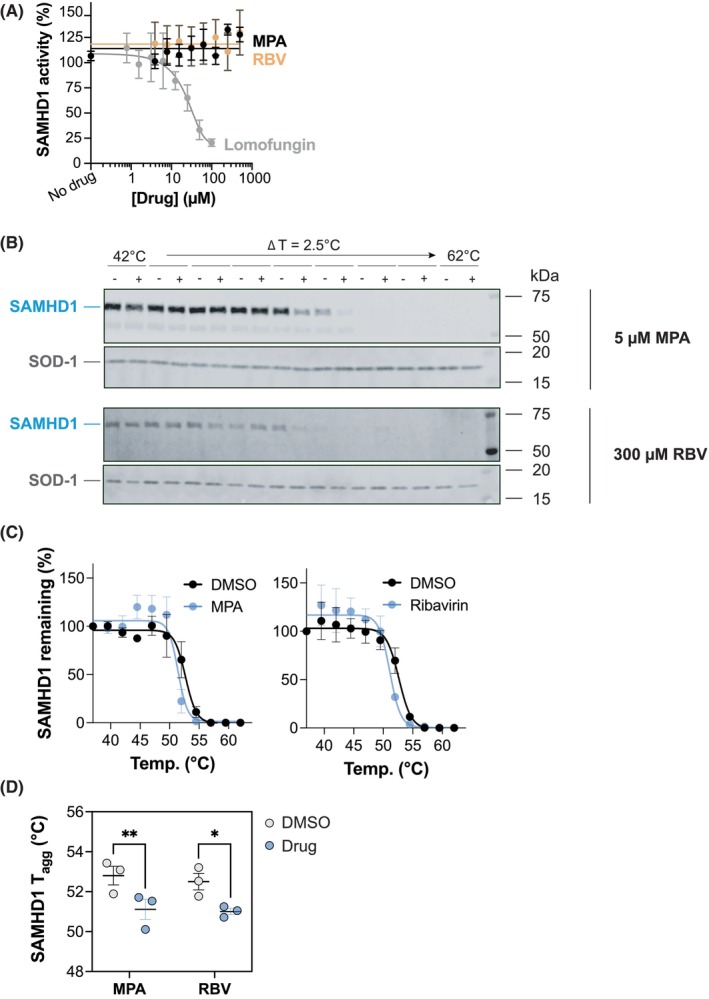
MPA and ribavirin do not inhibit SAMHD1 directly but treatment of cells with these compounds decreases the thermal stability of SAMHD1. (A) Recombinant SAMHD1 (0.35 μm) was incubated with a titration of mycophenolic acid (MPA), ribavirin (RBV) or lomofungin prior to addition of dGTP (25 μm) and measurement of dGTPase activity in the enzyme‐coupled activity assay. Mean values (*n* = 3, each performed with technical quadruplicates) plotted, error bars indicate SEM. (B, C) THP‐1 cells were incubated with either dimethyl sulfoxide (DMSO) or the indicated IMPDH inhibitor for 3 h at 37 °C before harvesting for cellular thermal shift assay (CETSA). Representative cropped immunoblot shown in (B) of three independent experiments, quantified and mean values (relative to SOD‐1 thermostable control) plotted in (C); error bars indicate SEM. (D) SAMHD1 thermal aggregation (*T*_agg_) values in MPA or RBV treated THP‐1 cells, as in (B). Mean values from three independent experiments plotted, error bars indicate SEM. Two‐tailed paired *t*‐test: *P < 0.05 and **P < 0.01.

First, we wanted to exclude that MPA and ribavirin are capable of directly inhibiting SAMHD1 activity, and thus, we tested these compounds against recombinant SAMHD1 protein in an enzyme‐coupled activity assay [25]. When MPA and ribavirin were assayed in combination with the SAMHD1 allosteric activator and substrate dGTP, no changes in SAMHD1 dGTP hydrolysis were observed even at concentrations up to 500 μm, whereas increasing doses of lomofungin, a positive control [11], led to dose‐dependent inhibition (Fig. 3A). These data demonstrate that the compounds MPA and ribavirin do not directly inhibit SAMHD1 dNTPase activity.

Both ribavirin and MPA can be metabolised inside cells, and thus to investigate whether drug metabolites could interact with SAMHD1, and/or perturb SAMHD1 protein stability, we employed cellular thermal shift assays (CETSA) [31]. This approach exploits ligand‐induced changes in protein melting, which we have previously validated as an applicable approach for evaluating SAMHD1 binders [15]. We incubated SAMHD1‐proficient THP‐1 cells with high concentrations (10‐fold IC_50_ values) of MPA or ribavirin, together with a DMSO control, for 3 h before collecting for CETSA (Fig. 3B,C). Thymidine treated cells were included as a positive control [15] (Fig. S5). Whilst thymidine exposure resulted in an increase in SAMHD1 thermal stability (Fig. S5), presumably through the triphosphate metabolite interacting with SAMHD1 allosteric and/or catalytic sites, both MPA and ribavirin exposed cells presented a decrease in SAMHD1 thermal stability (Fig. 3B–D). Similar results were obtained from 24‐h treatment with lower doses (around IC_50_ values) (Fig. S6). Drug‐induced changes in protein melting behaviour can be indicative of direct drug‐protein interaction, but also other events, including changes in protein‐interaction partners or oligomerisation status. These data indicate that treatment of cells with IMPDH inhibitors MPA and ribavirin, which do not inhibit SAMHD1 activity *in vitro*, do affect SAMHD1 protein stability in cells, however in a manner that is distinct from nucleotide ligands.

### 3.3 IMPDH inhibitors MPA and ribavirin deplete dGTP pools and expand dCTP pools in leukaemic cells

**Fig. 4:**
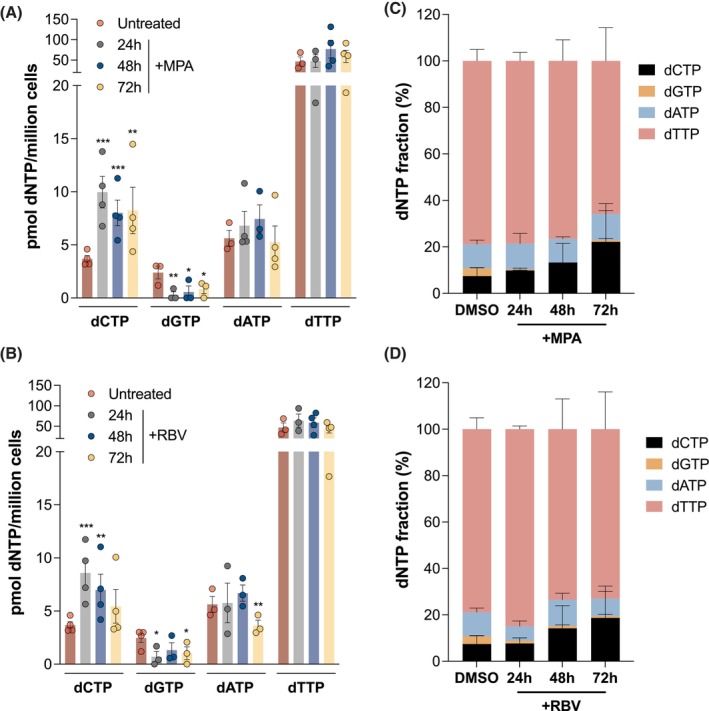
Quantification of dNTP pools in IMPDH inhibitor treated cells. THP‐1 cells were treated for 72 h with 0.5 μm mycophenolic acid (MPA) (A, C) or 25 μm ribavirin (RBV) (B, D). Extracts were collected at 24, 48 and 72 h post treatment, and deoxyribonucleoside triphosphate (dNTP) levels quantified by a polymerase‐based assay coupled to a click reaction. Absolute dNTP levels plotted in (A, B), dots represent mean values from experiments performed in duplicate (*n* = 3–4 independent experiments), error bars indicate SEM. Two‐tailed unpaired *t*‐test, *P < 0.05, **P < 0.01 and ***P < 0.001. Relative dNTP pool composition shown in (C, D), calculated using the total pmol of the four dNTPs as 100%. Data are presented as mean ± SEM.

We next sought to verify that MPA and ribavirin can inhibit IMPDH in leukaemic cells and thereby deplete guanine nucleoside triphosphates. SAMHD1‐proficient THP‐1 cells were treated with MPA and ribavirin for up to 72 h, and after each day, collected and dNTPs extracted and quantified. Both MPA and ribavirin significantly depleted dGTP levels throughout the course of the experiment (Fig. 4A,B), largely removing this species from the dNTP pool (Fig. 4C,D). Coinciding with dGTP depletion following MPA or ribavirin treatment, a significant expansion of the dCTP pool was observed in both cases which persisted for at least 72 h (Fig. 4). These data support that MPA and ribavirin inhibit IMPDH in leukaemic cells and thus can block the production of guanine nucleotides, but also highlight how this can result in an imbalance between purine and pyrimidine dNTP pools.

### 3.4 IMPDH inhibitor and cytarabine drug‐combination effect correlates with SAMHD1 abundance in haematological cancer cell models

**Fig. 5:**
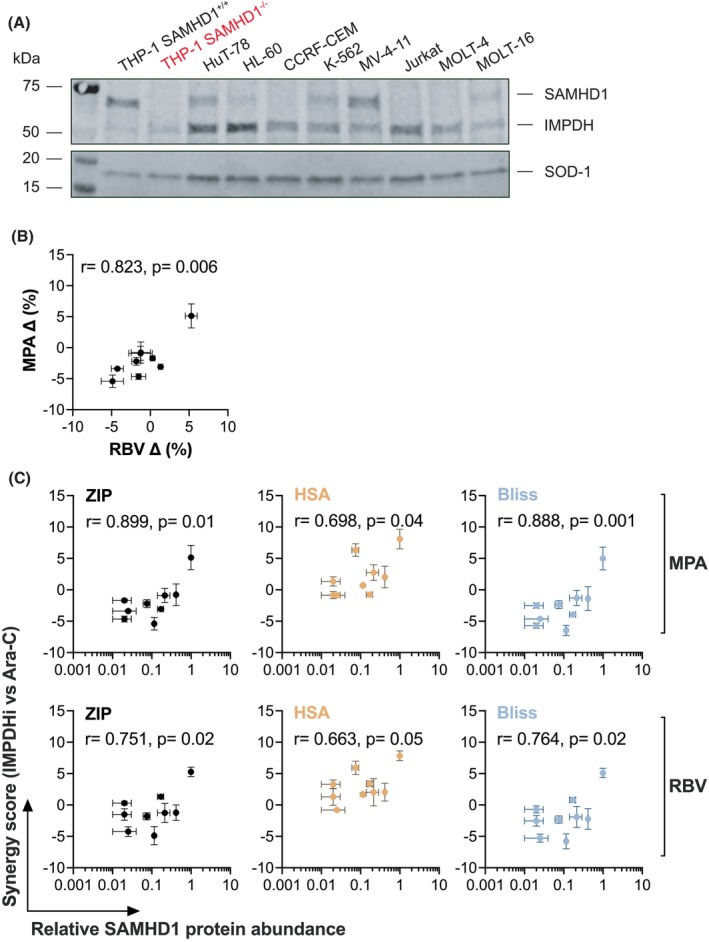
SAMHD1 protein abundance in blood cancer cell lines correlates with the extent of drug–drug interaction between cytarabine and IMPDH inhibitors. (A) Immunoblot analysis of lysates from a panel of haematological cancer cell lines. Representative cropped blot of two independent experiments. SOD‐1 was used as a loading control. (B) Correlation of ZIP synergy scores obtained upon combined treatment of mycophenolic acid (MPA) and cytarabine (ara‐C) *versus* ara‐C and ribavirin (RBV). Mean values with SD plotted from three independent experiments, each performed in duplicate. Pearson correlation with *P* value (two‐tailed) indicated. (C) Correlation of relative SAMHD1 protein abundance and synergy scores (derived from the indicated models) from combined treatment of ara‐C and MPA or ara‐C and RBV in a collection of haematological cancer cell lines. Mean abundance of SAMHD1 was calculated using SOD‐1 as a loading control and relative to THP‐1 expression from two independent experiments. Average delta scores from three independent experiments each with technical duplicates. Means values with SD plotted. Pearson correlations with *P* values (two‐tailed) indicated.

The SAMHD1 dependency of the drug–drug interaction between ara‐C and IMPDH inhibitors ribavirin or MPA has been observed thus far in cell models in which SAMHD1 expression has been ablated using CRISPR‐Cas9. Next, we wanted to interrogate this relationship in unperturbed cancer cell models and sought to determine whether the extent of this drug–drug interaction correlated with basal expression levels of SAMHD1. We assembled a panel of 9 haematological cancer cell lines with differing SAMHD1 protein levels (Fig. 5A) and, following determination of applicable monotherapy concentration ranges, subjected this cell line panel to dose matrices of ara‐C in combination with MPA or ribavirin in proliferation inhibition assays (Fig. S7) and calculated drug‐combination summary scores. Within this panel of cell lines, the drug‐combination scores derived from each of the IMPDH inhibitors correlated strongly with each other (Figs 5B, S8), supporting that these two chemically distinct molecules combined with ara‐C via a similar mechanism. When plotting the drug‐combination scores as a function of SAMHD1 protein abundance, we observed a strong and significant correlation between SAMHD1 abundance and the extent of the drug–drug interaction, independent of the synergy model used (Fig. 5C). Notably, and distinct from nonallosteric inhibitors of RNR [16], the correlation extends from a synergistic combination effect between ara‐C and IMPDH inhibitors in cell models with high basal SAMHD1 expression to a weakly antagonistic relationship in those cell models lacking basal SAMHD1 expression. Taken together, these data indicate that SAMHD1 protein levels dictate the extent of the drug–drug interaction between ara‐C and IMPDH inhibitors MPA and ribavirin in haematological cancer cell lines.

### 3.5 IMPDH inhibition potentiates cytarabine‐induced DNA damage and apoptosis

**Fig. 6:**
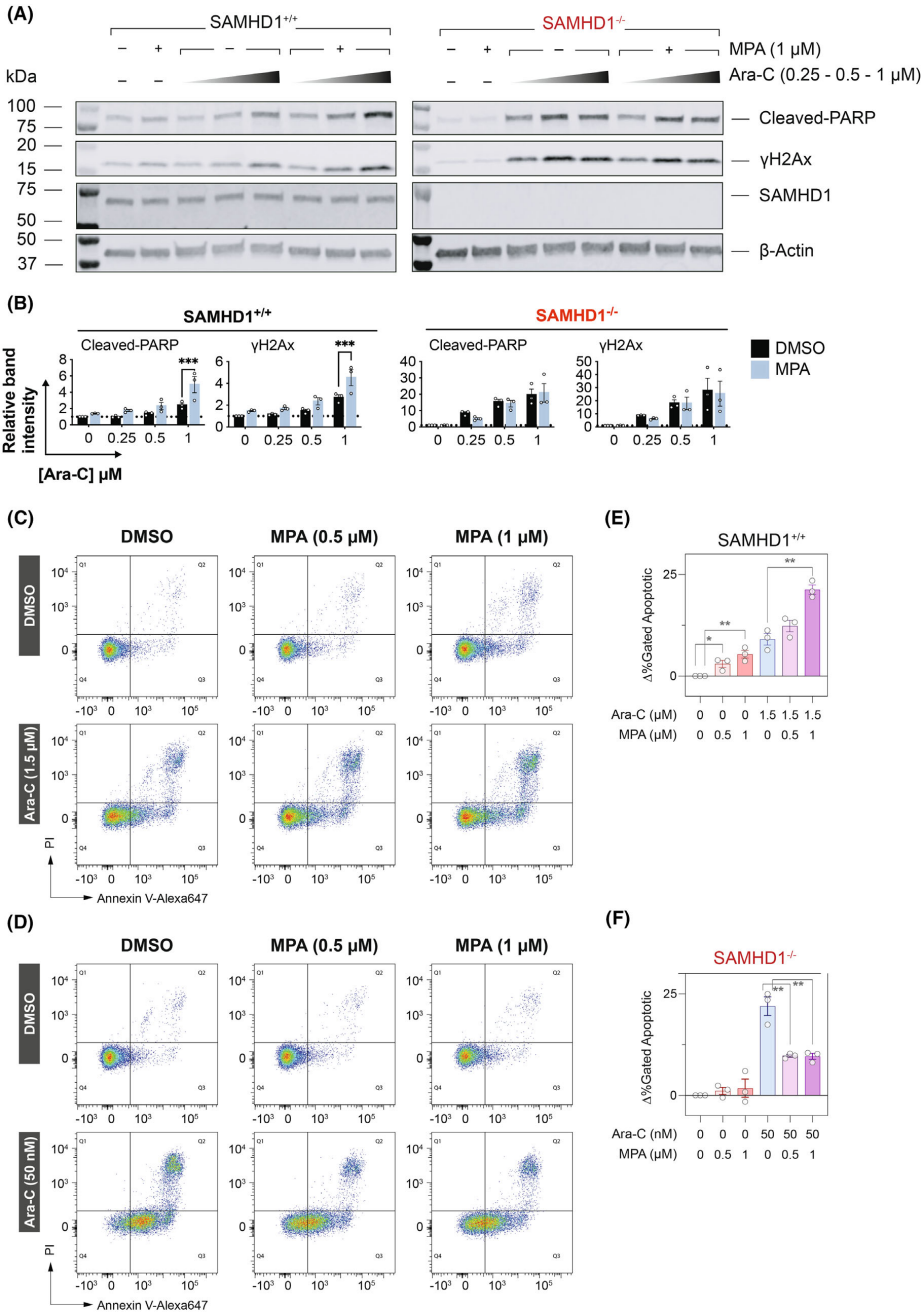
Analysis of DNA damage response and apoptotic signalling upon combined treatment with cytarabine and IMPDH inhibitor MPA. (A, B) *SAMHD1* ^+/+^ or *SAMHD1* ^−/−^ THP‐1 cells were cultured for 24 h with mycophenolic acid (MPA) (1 μm) and increasing concentrations of cytarabine (ara‐C) (0.25, 0.5 and 1 μm) before harvesting for immunoblot analysis with the indicated antibodies. Representative cropped immunoblot from 3 independent experiments shown in (A), mean band intensity relative to loading control plotted in (B), error bars indicate SEM. Two‐tailed unpaired *t*‐test, ***P < 0.001. (C–F) *SAMHD1* ^+/+^ (C, E) or *SAMHD1* ^−/−^ (D, F) THP‐1 cells were cultured with ara‐C (1.5 or 0.05 μm, respectively) or dimethyl sulfoxide (DMSO), alone or in combination with MPA (0.5 or 1 μm) for 48 h before apoptosis was assayed via Annexin V/PI staining followed by flow cytometry analysis. Representative flow cytometry plots shown (C, D) together with changes of Annexin‐V/PI double‐positive population compared with DMSO‐only group (∆% Gated), mean ∆% Gated of *n* = 3 independent experiments are plotted, together with values of individual experiments; error bars indicate SEM (E, F). Student's *t*‐tests (unpaired, two‐tailed) were performed across treatment groups, specifically between MPA‐ and DMSO‐treated groups, in the presence or absence of ara‐C, where asterisk signifies statistical significance (*P ≤ 0.05, **P ≤ 0.01).

The active triphosphate metabolite of ara‐C is a substrate for cellular replicases and is mis‐incorporated into genomic DNA resulting in DNA damage and apoptosis [3]. Next, we investigated whether treatment of leukaemic cells with IMPDH inhibitors MPA and ribavirin could potentiate ara‐C induced DNA damage and apoptotic signalling in a SAMHD1‐dependent manner. SAMHD1‐proficient and deficient THP‐1 cells were incubated with a titration of ara‐C in the absence or presence of MPA or ribavirin for 24 h before preparation of cell lysates and immunoblot analysis for DNA damage and apoptotic signalling. In the presence of SAMHD1, co‐treatment of ara‐C and MPA increased the abundance of active caspase product cleaved‐PARP and DNA damage signalling histone variant γH2AX (Fig. 6A,B). In the absence of SAMHD1, administration of ara‐C alone led to robust γH2AX and cleaved‐PARP signal, indicative that this phenotype is dependent upon SAMHD1 expression, which did not increase further with MPA addition (Fig. 6A,B). Similar results, albeit to a lower extent, were obtained with ribavirin (Fig. S9a,b). Next, in an orthogonal approach, we measured the proportion of apoptotic cells using Annexin V and PI staining coupled with flow cytometry detection (gating strategy shown in Fig. S10). Here, SAMHD1‐proficient and deficient THP‐1 cells were treated with MPA or ribavirin in the absence or presence of an ara‐C concentration that, as a monotherapy, led to an apoptotic population of 10–20% after 48 h (Figs 6C–F, S9c–f). In SAMHD1‐proficient cells, MPA treatment alone led to a small increase in apoptotic cells, but this MPA dose was capable of significantly augmenting the apoptotic population when combined with ara‐C (Fig. 6C,E). In contrast, in SAMHD1‐deficient cells, addition of MPA antagonised ara‐C induced apoptosis (Fig. 6D,F). Consistent with these findings, similar results were obtained with ribavirin (Fig. S9c–f). Taken together, these results show IMPDH inhibitors can modulate ara‐C induced DNA damage and apoptosis signalling in a SAMHD1‐dependent manner.

### 3.6 Deoxyguanosine supplementation reverts the effects of IMPDH inhibitor and cytarabine combination in SAMHD1‐expressing leukaemic cells

**Fig. 7:**
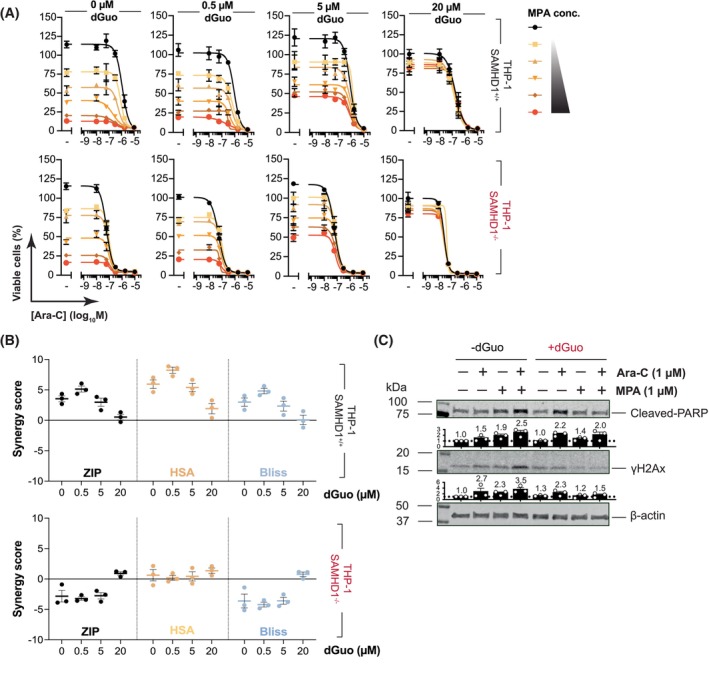
Deoxyguanosine supplementation reverts the effects of MPA in combination with cytarabine. (A) Proliferation inhibition analysis of cytarabine (ara‐C, 0.01–10 μm) and mycophenolic acid (MPA, 0.5–5 μm) treated *SAMHD1* ^+/+^ and *SAMHD1* ^−/−^ THP‐1 cells supplemented with the indicated concentrations of deoxyguanosine (dGuo). Mean values plotted ± SEM from three independent experiments each performed in duplicate. (B) Drug synergy plots for ara‐C and MPA derived from treatments in (A). Each data point indicates an average delta score from the indicated synergy models from a single dose–response matrix experiment performed in duplicate. *N* = 3, mean ± SEM represented with horizontal and vertical bars, respectively. (C) *SAMHD1* ^+/+^ THP‐1 cells were cultured for 24 h with the indicated doses of ara‐C and/or MPA with or without dGuo (5 μm) before harvesting for immunoblot analysis with the indicated antibodies. Representative cropped immunoblot from three independent experiments shown. Mean band intensity (*n* = 3) relative to loading control and normalised to untreated plotted below, error bars indicate SEM.

Thus far, we have identified IMPDH inhibitors MPA and ribavirin as two compounds capable of eliciting SAMHD1‐dependent sensitisation to ara‐C, and our data support this is not via direct inhibition of SAMHD1 but rather by inhibition of IMPDH and depletion of guanine nucleotides. To further test this, we reasoned that supplementation of culture media with cell‐permeable dGTP precursor deoxyguanosine (dGuo) would restore intracellular dGTP levels thereby circumventing IMPDH inhibition and abrogate the SAMHD1‐dependent phenotypes in ara‐C exposed leukaemic cells. First, we tested a dose range of dGuo in cell proliferation inhibition assays and observed that treatments with 50 μm or higher dGuo reduced cell proliferation both in SAMHD1‐proficient and SAMHD1‐deficient THP‐1 cells. Consistent with previous reports, cells lacking SAMHD1 were more sensitive to dGuo treatment and subsequent dGTP overload [32, 33] (Fig. S11a). We therefore decided to evaluate ara‐C synergy with IMPDH inhibitors MPA or ribavirin when up to 20 μm dGuo was added to the media, as the concentrations of dGuo used in the experiment were all noncytotoxic (Fig. S11b). Increasing concentrations of dGuo restored cell viability when combined with cytarabine and MPA or ribavirin (Figs 7A, S12a), but also led to a dose‐dependent reduction in drug‐combination effect between the IMPDH inhibitors and cytarabine in SAMHD1‐proficient cells (Figs 7B, S12b). In SAMHD1 deficient cells, no substantial changes in ara‐C sensitisation were observed at any of the dGuo doses used in the experiments (Figs 7A, S12a), suggesting that indeed the phenotype depends on SAMHD1 expression, and furthermore, the drug‐combination effect was distinct from SAMHD1‐proficient cells (Figs 7A,B, S12a,b).

We next evaluated the effects of dGuo supplementation upon combined treatment with IMPDH inhibitors and ara‐C on the activation of DNA damage and apoptotic signalling. In line with our earlier data, co‐treatment of IMPDH inhibitor and ara‐C led to an increase in DNA damage (γH2AX) and apoptotic signalling (cleaved‐PARP) compared with either treatment alone (Figs 7C, S12c). Supporting our hypothesis, supplementation of cell medium with dGuo reduced these signalling cascades (Figs 7C, S12c). Taken together, these data indicate the SAMHD1‐dependent synergy and DNA damage/apoptotic signalling observed when combining ara‐C and IMPDH inhibitors is due to reduction of dGTP biosynthesis.

## 4 Discussion

The deoxycytidine analogue ara‐C is a central pillar of AML therapy and lack of response to this agent is a principal cause of treatment failure and relapse [2]. A major determinant of ara‐C therapy efficacy in AML is the dNTP hydrolase SAMHD1 [4, 5] and accordingly, this enzyme constitutes an attractive therapeutic target to improve treatment responses [8]. Various approaches have been taken to target SAMHD1 (reviewed in [34]) but the vast majority of these strategies, specifically those which aim to interfere with SAMHD1 activity via direct interaction, are currently restricted to the preclinical setting. The exception is the indirect pharmacological targeting of SAMHD1 with inhibitors of RNR, which were identified via an unbiased phenotypic approach [16] and are now being evaluated in the clinic [20]. This approach exploits the allosteric regulation of SAMHD1 hydrolytic activity by dNTPs, and current data support a model in which RNR inhibition results in a dNTP pool perturbation, specifically a reversal of the dATP‐to‐dCTP ratio, which alters the allosteric activator of SAMHD1 at AS2 leading to reduced ara‐CTPase activity [16]. This prompted the question of whether other dNTP pool perturbations could affect SAMHD1 activity in cells and given the plethora of existing drugs targeting nucleotide biosynthesis, could offer additional potential drug‐repurposing approaches to indirectly target SAMHD1. Thus, in the present study we screened a collection of nucleotide biosynthesis inhibitors for their ability to elicit SAMHD1‐dependent synergy with ara‐C and identified inhibitors of IMPDH, the enzyme responsible for catalysing the rate‐limiting step in guanine nucleotide production. Accordingly, we show IMPDH inhibitors ribavirin and MPA can efficiently deplete dGTP pools in leukaemic cells and sensitise leukaemic cells with high SAMHD1 expression to ara‐C in a SAMHD1‐dependent manner. Furthermore, the SAMHD1‐dependent effects on ara‐C efficacy in IMPDH inhibitor treated cells could be reversed by supplementation of media with cell‐permeable dGTP precursor dGuo, pinpointing dGTP depletion as a key contributing factor.

Guanine nucleotides are critical for the activation of SAMHD1 [19]. AS1 specifically requires binding of a guanine nucleotide for activation, with GTP being the likely physiological activator owing to high micromolar concentrations in cells [35]. AS2 binds dNTPs, and so dGTP can bind at this site in addition to binding AS1, given it is also a guanine nucleotide. Thus, targeting of guanine nucleotide biosynthesis via the rate‐limiting enzyme IMPDH could potentially disrupt allosteric activation of SAMHD1 via both AS1 and AS2. Interestingly, in addition to the efficient depletion of dGTP pools we observed in IMPDH inhibitor treated leukaemic cells, we consistently observed expansion of dCTP pools. There are several possible explanations which are not mutually exclusive. Firstly, dCTP pool expansion could be mediated by allosteric regulation of RNR, given dGTP is the allosteric stimulator of ADP reduction [36], thus lack of dGTP would lead to accumulation of ADP and subsequently ATP thereby stimulating CDP reduction to dCDP and the resulting accumulation of dCTP. However, this would be expected to coincide with dATP depletion which was only observed at longer IMPDH inhibitor treatments. In addition, nucleoside salvage via deoxycytidine kinase (dCK) could contribute to dCTP pool expansion as we have reported previously with regards to RNR inhibitors [16], or could potentially buffer dATP pools. Considering dCTP pool expansion coincides with dGTP pool depletion, delineating whether the precise molecular mechanism of apparent SAMHD1 activity suppression is distinct from that of nonallosteric RNR inhibitors is difficult. Treatment of leukaemic cells with nonallosteric RNR inhibitors resulted in an inversion of the dCTP‐to‐dATP ratio, and given the extent of dCTP pool expansion observed following IMPDH inhibitor treatment, the dCTP pool does exceed that of the dATP pool. Thus, a similar AS2‐directed mechanism of dCTP‐activated SAMHD1 being a poor ara‐CTPase could also be the cause here. A critical difference is that the SAMHD1‐dependent sensitisation to ara‐C observed with IMPDH inhibitors can be reversed by dGuo supplementation, whilst this has not been documented for RNR inhibition. Supplementation of dGuo would restore dGTP pools [37, 38] but it could well be that restoration of intracellular dGTP levels leads to a normalisation of dCTP pools. Recent data from in‐depth biochemical studies of SAMHD1 allosteric activation [39] provide potential support for a unified mode‐of‐action between inhibitors of RNR and IMPDH with respect to indirect targeting of SAMHD1. With recombinant SAMHD1 protein, this study demonstrated that efficient depletion of pyrimidine nucleotides is dependent upon the availability of purine nucleotides [39]. Thus, our observations here with IMPDH inhibitors and in our previous study with RNR inhibitors [16] in leukaemic cells, in which purine (dATP or dGTP) nucleotides are depleted but pyrimidine (dCTP) nucleotides expand, which increases the efficacy of a pyrimidine analogue ara‐C, are consistent with the proposed mechanism. Further studies will need to focus upon elucidating this.

IMPDH inhibitors have long been considered as anticancer agents, but their efficacy in the clinic remains to be established [28]. Focusing upon AML, several studies have documented the antileukaemic effects of different IMPDH inhibitors in preclinical models [28, 40, 41, 42], and a recent study highlighted a particular utility of IMPDH inhibition in AMLs driven by MLL‐fusion [43]. Several mechanisms underpinning the antileukaemic activity of IMPDH inhibitors have been described, including induction of differentiation and cell death mediated by guanine nucleotide depletion [40, 41, 43] and, in the case of ribavirin, an IMPDH‐independent function through inhibition of the eukaryotic translation initiation factor 4E (eIF4E) oncogene [44]. Owing to overexpression of eIF4E in several AML subtypes, ribavirin has been investigated clinically in AML as a monotherapy [45] and subsequently in combination with low‐dose ara‐C [46]. The latter study was based on observations that ribavirin can enhance the antileukaemic effect of ara‐C in primary AML samples [47], in line with our observations in AML cell models. What is clear from these studies and our own is that IMPDH inhibitors possess monotherapy antileukaemic efficacy, and a particular benefit of using these drugs as an indirect approach to target SAMHD1 is the combination of individually active drugs, which is a critical consideration when designing combination therapies [48].

This study has several limitations. Whilst we could recapitulate the findings of our study with two chemically distinct molecules that target IMPDH, these compounds are not chemical probes and will have additional cellular targets. When defining the underpinning mechanism of indirect SAMHD1 targeting observed here, chemical probes or genetic approaches to specifically perturb IMPDH function would be important. In addition, whilst we have demonstrated the antileukaemic activity of IMPDH inhibitors and ara‐C in established cell models, future studies should focus upon more complex preclinical cancer models.

## 5 Conclusion

In summary, here we present evidence that the approved drugs MPA and ribavirin—which target IMPDH, the rate‐limiting enzyme in guanine nucleotide production—imbalance dNTP pools in leukaemic cells and thereby indirectly suppress the ara‐CTP hydrolytic activity of SAMHD1. We thus propose that repurposing of IMPDH inhibitors, and combining these agents with ara‐C, could have potential utility in improving AML treatment.

## Conflict of interest

The authors declare no conflict of interest.

## Author contributions

MY‐C, NH and SGR contributed to the conceptualisation and writing—review and editing. MY‐C, CD, CE, SKF, FMH, YK, IL and SMZ contributed to the investigation. MY‐C and SGR contributed to the methodology and writing—original draft. MY‐C, SMZ and SGR contributed to the formal analysis and visualisation. MY‐C, IL, SMZ, NH and SGR contributed to the funding acquisition and supervision.

## Supporting information


**Fig. S1.** Drug synergy plots for ara‐C and the indicated nucleotide biosynthesis inhibitor in THP‐1 SAMHD1‐proficient (SAMHD1^+/+^) or ‐deficient (SAMHD1^−/−^) cells.
**Fig. S2.** Dose–response curves from nucleotide biosynthesis inhibitor and cytarabine drug combination screen in SAMHD1^+/+^ and SAMHD1^−/−^ THP‐1 cells.
**Fig. S3.** Drug synergy plots for ara‐C and the indicated nucleotide biosynthesis inhibitor in HuT‐78 and HL‐60 SAMHD1‐proficient (SAMHD1^+/+^) or ‐deficient (SAMHD1^−/−^) cell line pairs.
**Fig. S4.** Dose–response curves from nucleotide biosynthesis inhibitor and cytarabine drug combination screen in SAMHD1^+/+^ and SAMHD1^−/−^ HuT‐78 and HL‐60 cells.
**Fig. S5.** SAMHD1 CETSA in thymidine treated cells.
**Fig. S6.** SAMHD1 CETSA in cells exposed to mycophenolic acid or ribavirin for 24 h.
**Fig. S7.** Dose–response curves from IMPDH inhibitor and cytarabine drug combination screen in a panel of haematological cell lines.
**Fig. S8.** Correlation of IMPDH inhibitor vs cytarabine synergy scores.
**Fig. S9.** Activation of DNA damage and apoptotic signalling upon combined treatment with cytarabine and ribavirin.
**Fig. S10.** Flow cytometry gating strategy.
**Fig. S11.** Titration of deoxyguanosine and cell viability measurements.
**Fig. S12.** Effect of deoxyguanosine supplementation upon ribavirin and cytarabine combination treatment.

## Data Availability

The data that support the findings of this study are available from the corresponding author upon request (Sean Rudd, sean.rudd@scilifelab.se).
