# Supplementary material for: IMPDH inhibition enhances cytarabine efficacy in SAMHD1‐expressing leukaemia cells via guanine nucleotide depletion
Source: Mol Oncol. 2026 Feb 19;20(11):2936–54. doi: 10.1002/1878-0261.70226 (PMC13398360; doi:10.1002/1878-0261.70226)
Supplement: Supplementary file 1 [file MOL2-20-2936-s001.pdf]

Supplementary Figures S1 – S12

Figure S1

a

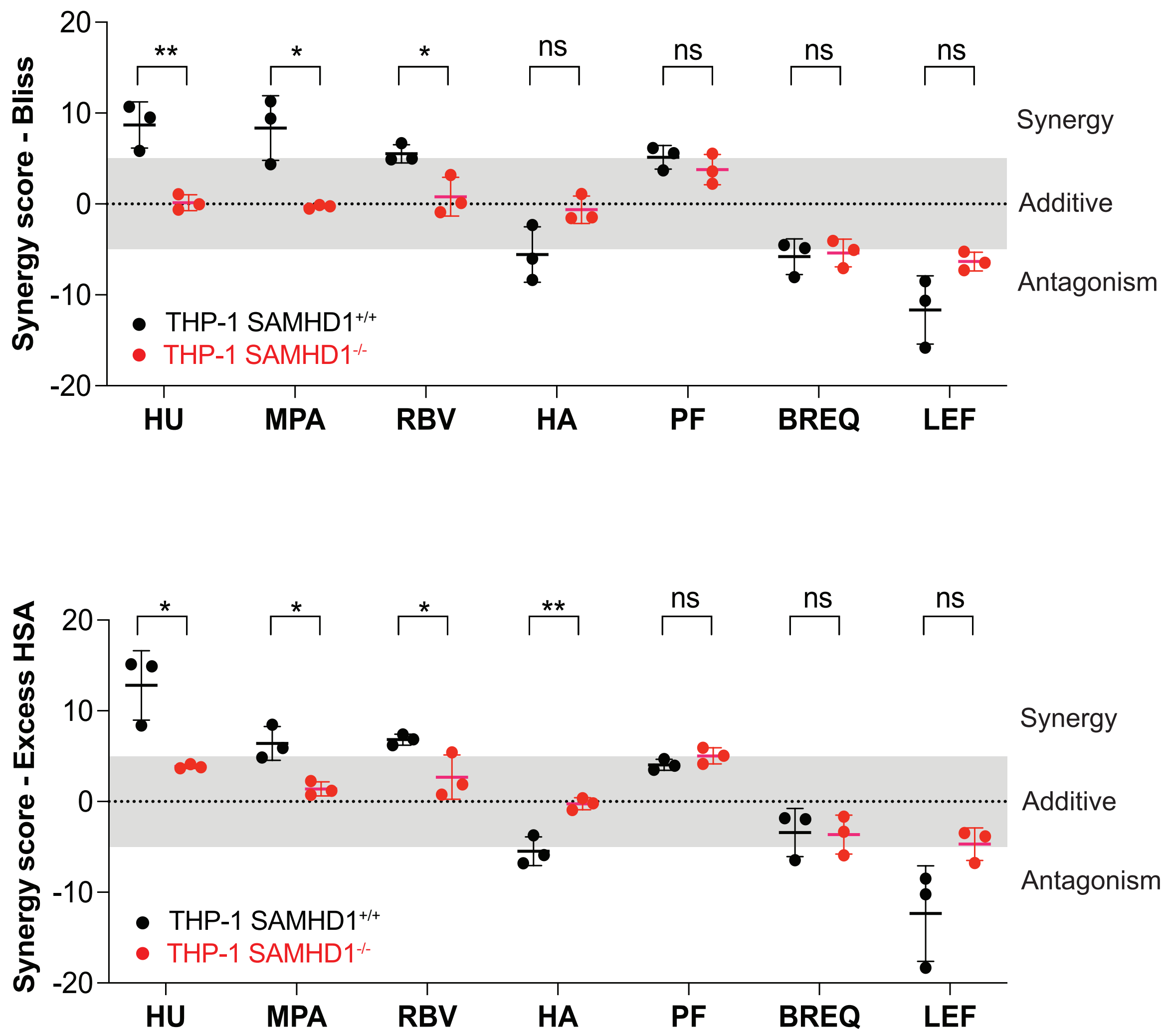

Synergy score - Bliss
20
10
0
-10
-20
**
*
*
ns
ns
ns
ns
Synergy
Additive
Antagonism
THP-1 SAMHD1+/+
THP-1 SAMHD1-/-
HU
MPA
RBV
HA
PF
BREQ
LEF

b

Synergy score - Excess HSA
20
10
0
-10
-20
*
*
*
**
ns
ns
ns
Synergy
Additive
Antagonism
THP-1 SAMHD1+/+
THP-1 SAMHD1-/-
HU
MPA
RBV
HA
PF
BREQ
LEF

Figure S1. Drug synergy plots for ara-C and the indicated nucleotide biosynthesis inhibitor in THP-1 SAMHD1-proficient (SAMHD1$^{+/+}$) or -deficient (SAMHD1$^{-/-}$) cells.

Each data point indicates an average synergy score from a single dose–response matrix experiment performed in duplicate. Synergy scores derived from the Bliss model (a) and HSA model (b) using the viability matrix as input. Synergy scores >5 relates to synergy, <5 indicates antagonism and between -5 and 5 is considered as additive. A total of three independent experiments were performed in technical duplicates and mean ± SD is represented with horizontal and vertical bars, respectively. Two-tailed unpaired t-test: *p<0.05 and **p<0.01. Abbreviations: HU (hydroxyurea), MPA (mycophenolic acid), RBV (ribavirin), HA (hadacidin), PF (pyrazofurin), BREQ (brequinar) and LEF (leflunomide).

Figure S2

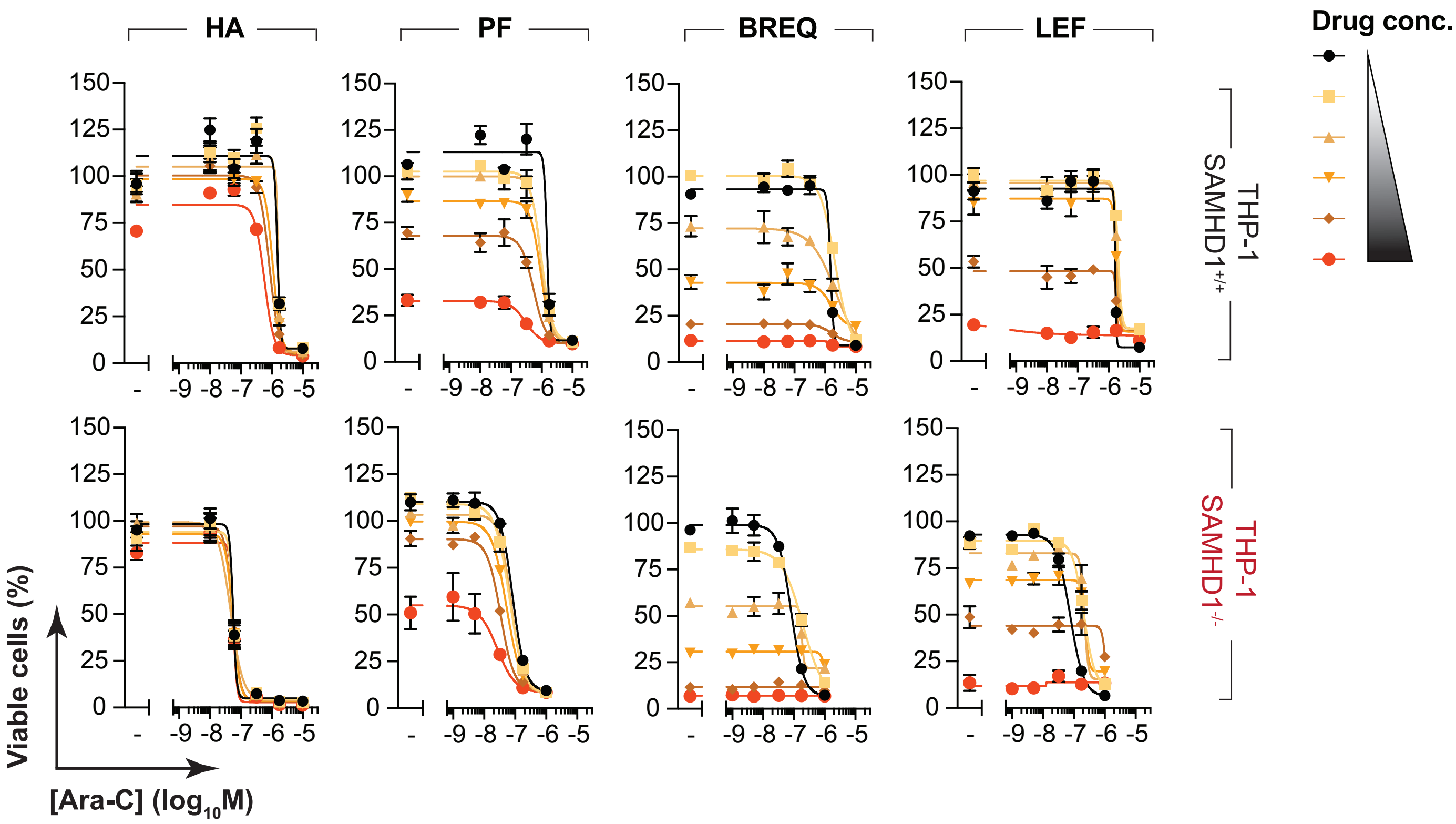

HA
PF
BREQ
LEF
Drug conc.
THP-1 SAMHD1+/+
THP-1 SAMHD1-/-
Viable cells (%)
[Ara-C] (log10M)
150
125
100
75
50
25
0
-
-9 -8 -7 -6 -5

Figure S2. Dose-response curves from nucleotide biosynthesis inhibitor and cytarabine drug combination screen in SAMHD1$^{+/+}$ and SAMHD1$^{-/-}$ THP-1 cells. Cell viability measurements via resazurin reduction assay following 4-day treatment with cytarabine (ara-C) vs nucleotide biosynthesis inhibitors. Drugs: hadacidin (HA, 10-450 μM); pyrazofurin (PF, 5-30 μM); brequinar (BREQ, 100-300 μM); leflunomide (LEF, 145-400 μM); ara-C (SAMHD1$^{+/+}$ cells, 0.01-10 μM; SAMHD1$^{-/-}$ cells, 0.001-1 μM). Plot displays mean from 2-3 independent experiments (n=3 HA; n=3 PF; n=3 BREQ; n=2-3 LEF) performed in technical duplicates, error bars represent SEM.

Figure S3

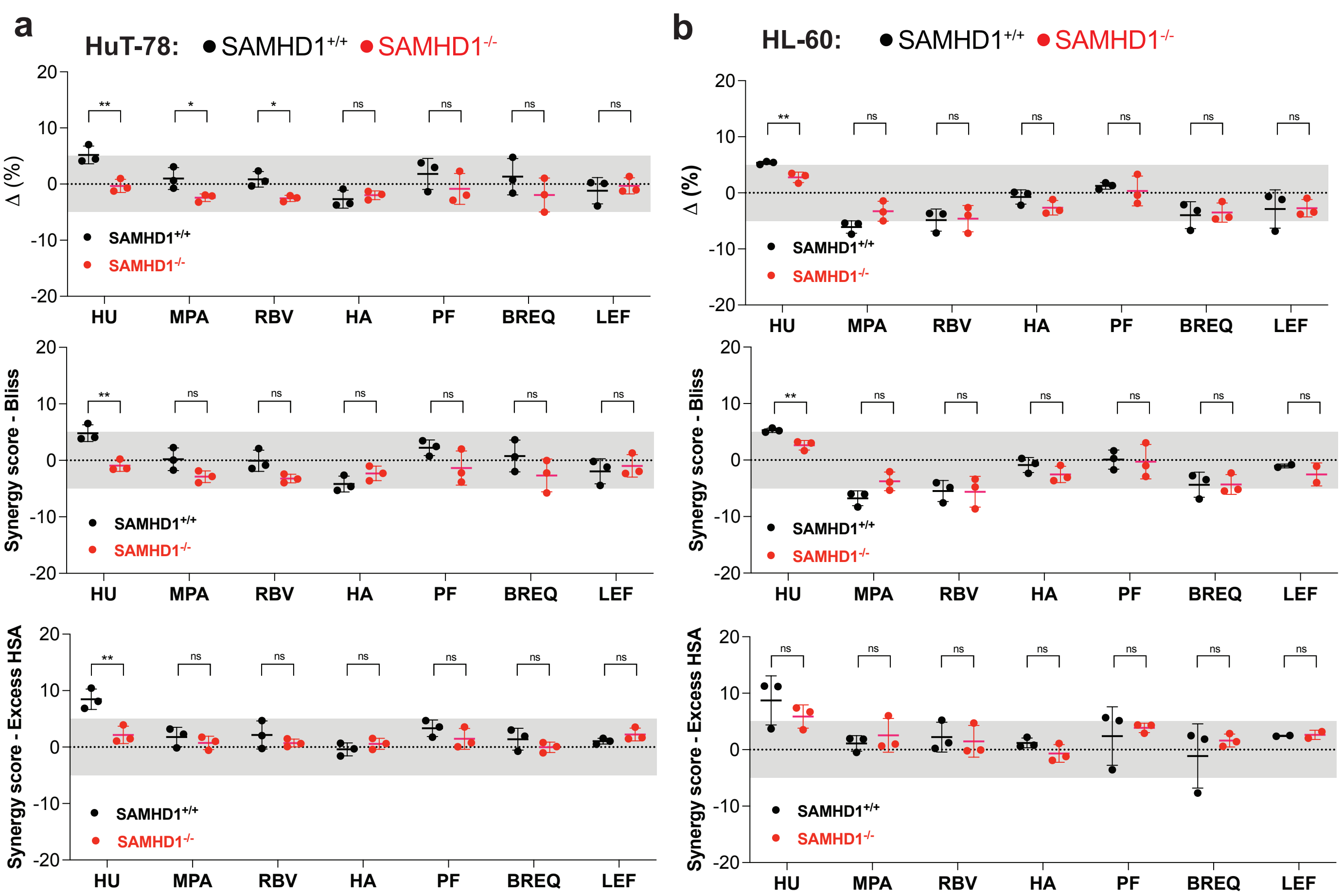
a
HuT-78: ● SAMHD1+/+ ● SAMHD1-/-
Δ (%)
Synergy score - Bliss
Synergy score - Excess HSA
SAMHD1+/+
SAMHD1-/-
HU
MPA
RBV
HA
PF
BREQ
LEF
**
*
ns
b
HL-60: ● SAMHD1+/+ ● SAMHD1-/-

Figure S3. Drug synergy plots for ara-C and the indicated nucleotide biosynthesis inhibitor in HuT-78 and HL-60 SAMHD1-proficient ($SAMHD1^{+/+}$) or -deficient ($SAMHD1^{-/-}$) cell line pairs.

Data from HuT-78 shown in (a) and HL-60 in (b). Each data point indicates an average synergy score from a single dose–response matrix experiment performed in duplicate. Synergy scores derived from the ZIP model, Bliss model, and HSA model – as indicated – using the viability matrix as input. Synergy scores >5 relates to synergy, <5 indicates antagonism and between -5 and 5 is considered as additive. A total of three independent experiments were performed in technical duplicates and mean ± SD is represented with horizontal and vertical bars, respectively. Two-tailed unpaired t-test: *p<0.05 and **p<0.01. Abbreviations: HU (hydroxyurea), MPA (mycophenolic acid), RBV (ribavirin), HA (hadacidin), PF (pyrazofurin), BREQ (brequinar) and LEF (leflunomide).

Figure S4

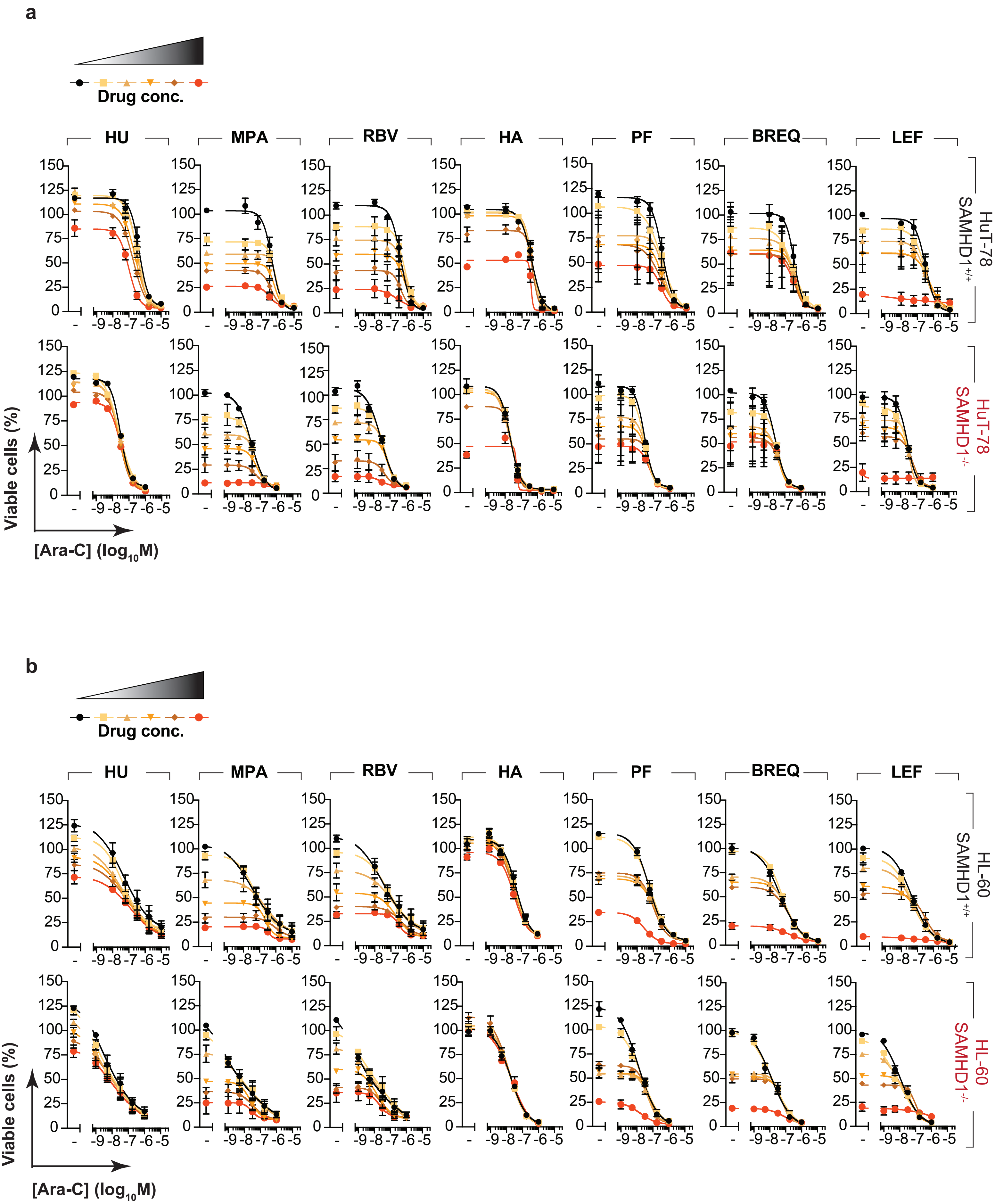
a
Drug conc.
HU
MPA
RBV
HA
PF
BREQ
LEF
HuT-78 SAMHD1+/+
HuT-78 SAMHD1-/-
Viable cells (%)
[Ara-C] (log10M)
b
Drug conc.
HU
MPA
RBV
HA
PF
BREQ
LEF
HL-60 SAMHD1+/+
HL-60 SAMHD1-/-
Viable cells (%)
[Ara-C] (log10M)

Figure S4. Dose-response curves from nucleotide biosynthesis inhibitor and cytarabine drug combination screen in SAMHD1$^{+/+}$ and SAMHD1$^{-/-}$ HuT-78 and HL-60 cells.

(**a**) Cell viability measurements via resazurin reduction assay following 4-day treatment with cytarabine (ara-C) vs nucleotide biosynthesis inhibitors in SAMHD1$^{+/+}$ and SAMHD1$^{-/-}$ HuT-78 cells. Drugs: hydroxyurea (HU, 5-150 μM); mycophenolic acid (MPA, 0.15-1.5 μM); ribavirin (RBV, 5-150 μM); hadacidin (HA, 10-450 μM); pyrazofurin (PF, 0.01-10 μM); brequinar (BREQ, 0.05-5 μM); leflunomide (LEF, 10-300 μM); ara-C (SAMHD1$^{+/+}$ cells, 0.01-10 μM; SAMHD1$^{-/-}$ cells, 0.001-1 μM). Values represent mean ± SEM from 2-3 independent experiments (n=3 HU; n=3 MPA; n=3 RBV; n=3 HA; n=2-3 PF; n=2 BREQ; n=2 LEF) performed in technical duplicates.

(b) Cell viability measurements via resazurin reduction assay following 4-day treatment with cytarabine (ara-C) vs nucleotide biosynthesis inhibitors in SAMHD1$^{+/+}$ and SAMHD1$^{-/-}$ HL-60 cells. Drugs: hydroxyurea (HU, 10-100 μM); mycophenolic acid (MPA, 0.1-2 μM); ribavirin (RBV, 5-100 μM); hadacidin (HA, 10-450 μM); pyrazofurin (PF, 0.01-30 μM); brequinar (BREQ, 0.01-150 μM); leflunomide (LEF, 5-300μM); ara-C (SAMHD1$^{+/+}$ cells, 0.01-10 μM; SAMHD1$^{-/-}$ cells, 0.001-1 μM). Values represent mean ± SEM from 2-3 independent experiments (n=3 HU, n=2-3 MPA, n=2-3 RBV; n=3 HA; n=2-3 PF; n=2-3 BREQ; n=2-3 LEF) performed in technical duplicates.

Figure S5

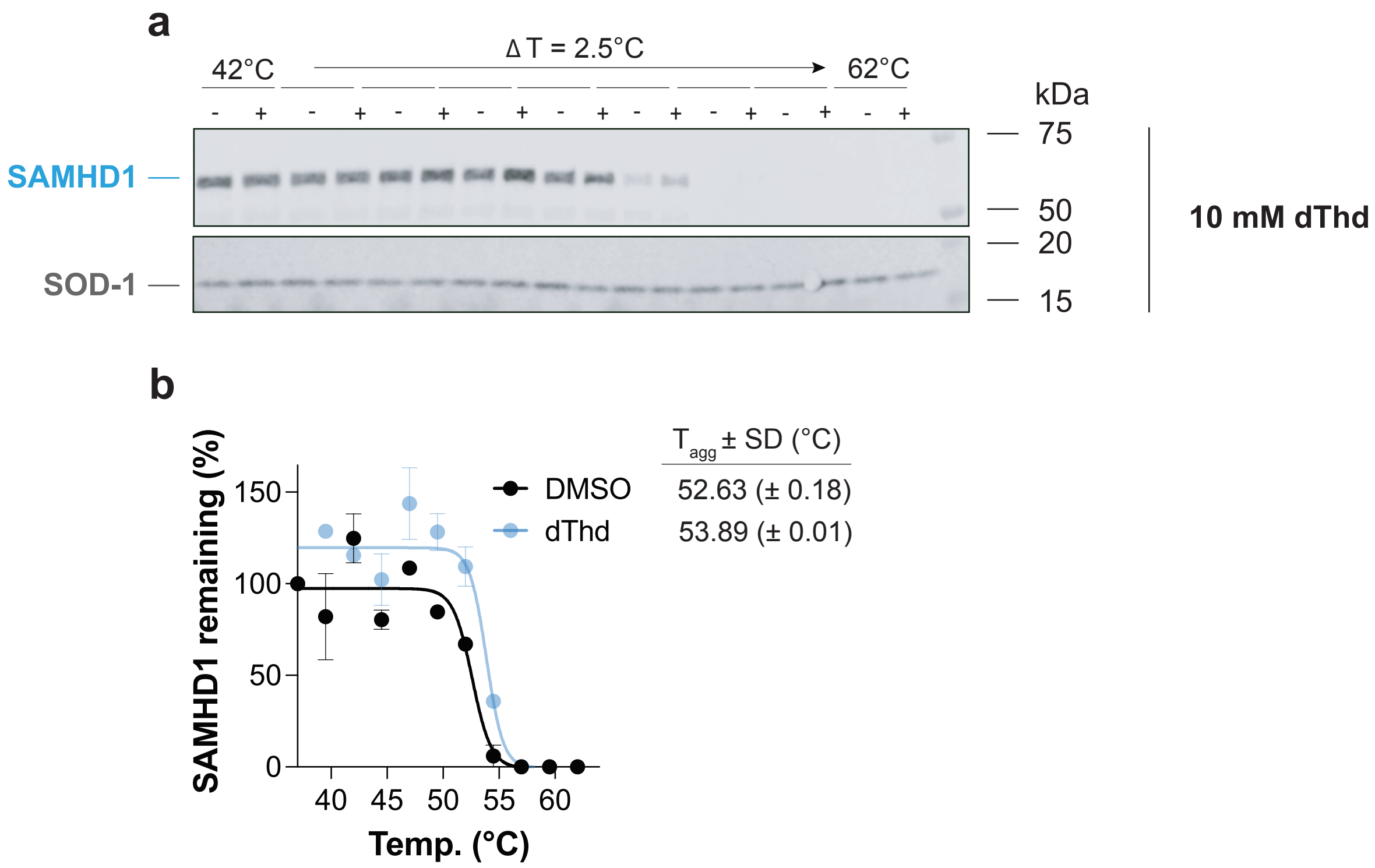

a
42°C
ΔT = 2.5°C
62°C
kDa
- + - + - + - + - + - + - + - + - + - +
SAMHD1
SOD-1
75
50
20
15
10 mM dThd
b
SAMHD1 remaining (%)
150
100
50
0
40 45 50 55 60
Temp. (°C)
DMSO
dThd
T agg ± SD (°C)
52.63 (± 0.18)
53.89 (± 0.01)

Figure S5. SAMHD1 CETSA in thymidine treated cells.

THP-1 cells were incubated with DMSO or thymidine (dThd) for 3 hours at 37 °C before harvesting for CETSA. Representative cropped immunoblot shown in (a) of two independent experiments, quantified and mean values (relative to SOD-1 thermostable control) plotted in (b), error bars indicate SEM. Mean thermal aggregation (T_{agg}) values shown.

Figure S6

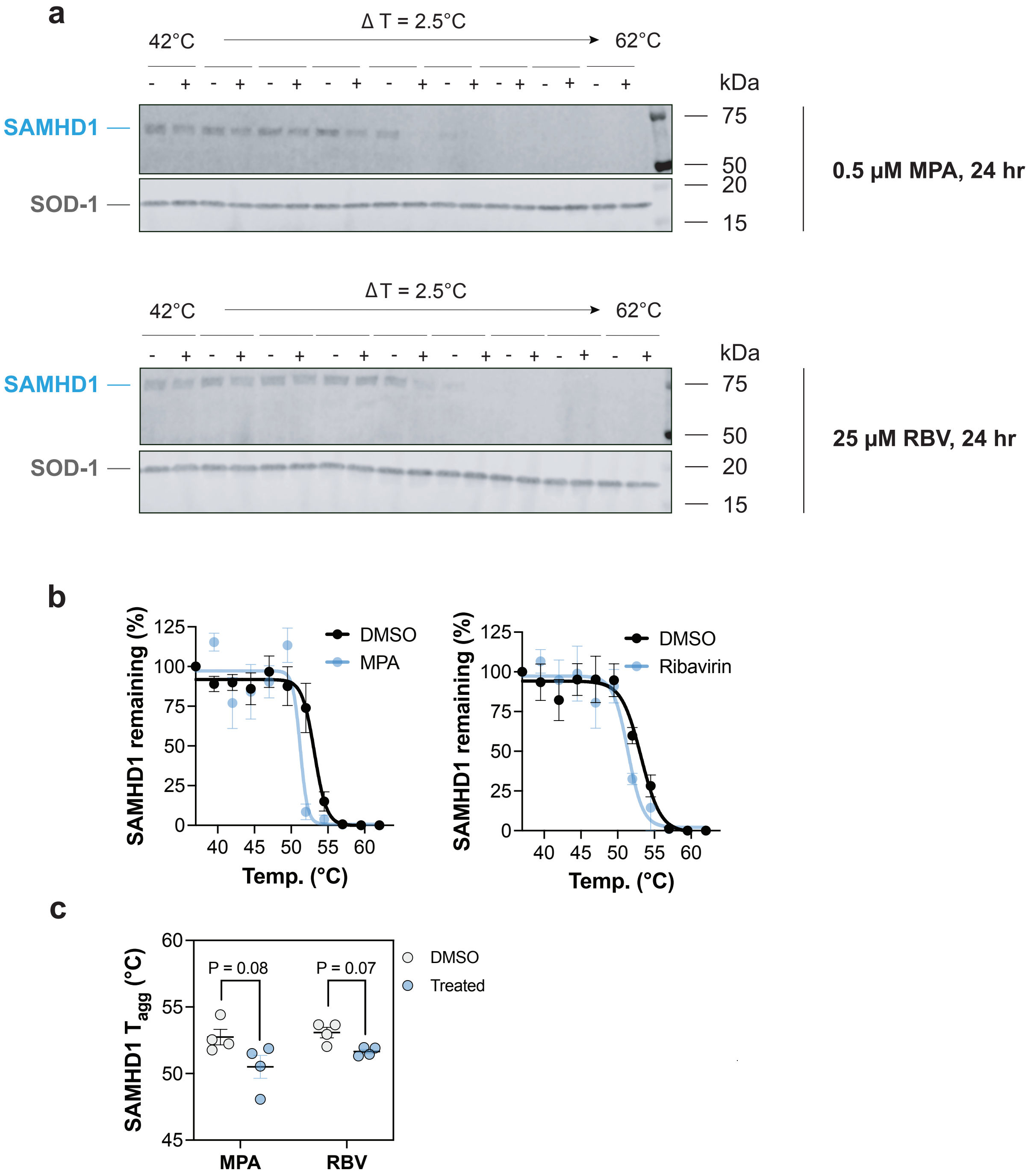
a
ΔT = 2.5°C
42°C
62°C
kDa
SAMHD1
SOD-1
75
50
20
15
0.5 μM MPA, 24 hr
25 μM RBV, 24 hr
b
SAMHD1 remaining (%)
Temp. (°C)
DMSO
MPA
Ribavirin
c
SAMHD1 Tagg (°C)
P = 0.08
P = 0.07
DMSO
Treated
MPA
RBV

Figure S6. SAMHD1 CETSA in cells exposed to mycophenolic acid or ribavirin for 24 hours.
(**a, b**) THP-1 cells were incubated with either DMSO or the indicated IMPDH inhibitor for 24 hours at 37 °C before harvesting for CETSA. Representative cropped immunoblot shown in (b) of four independent experiments, quantified and mean values (relative to SOD-1 thermostable control) plotted in (b), error bars indicate SEM.
(**c**) SAMHD1 thermal aggregation (T_{agg}) values in MPA or RBV treated THP-1 cells, as in (a). Mean values from four independent experiments plotted. Two-tailed paired t-test.

Figure S7

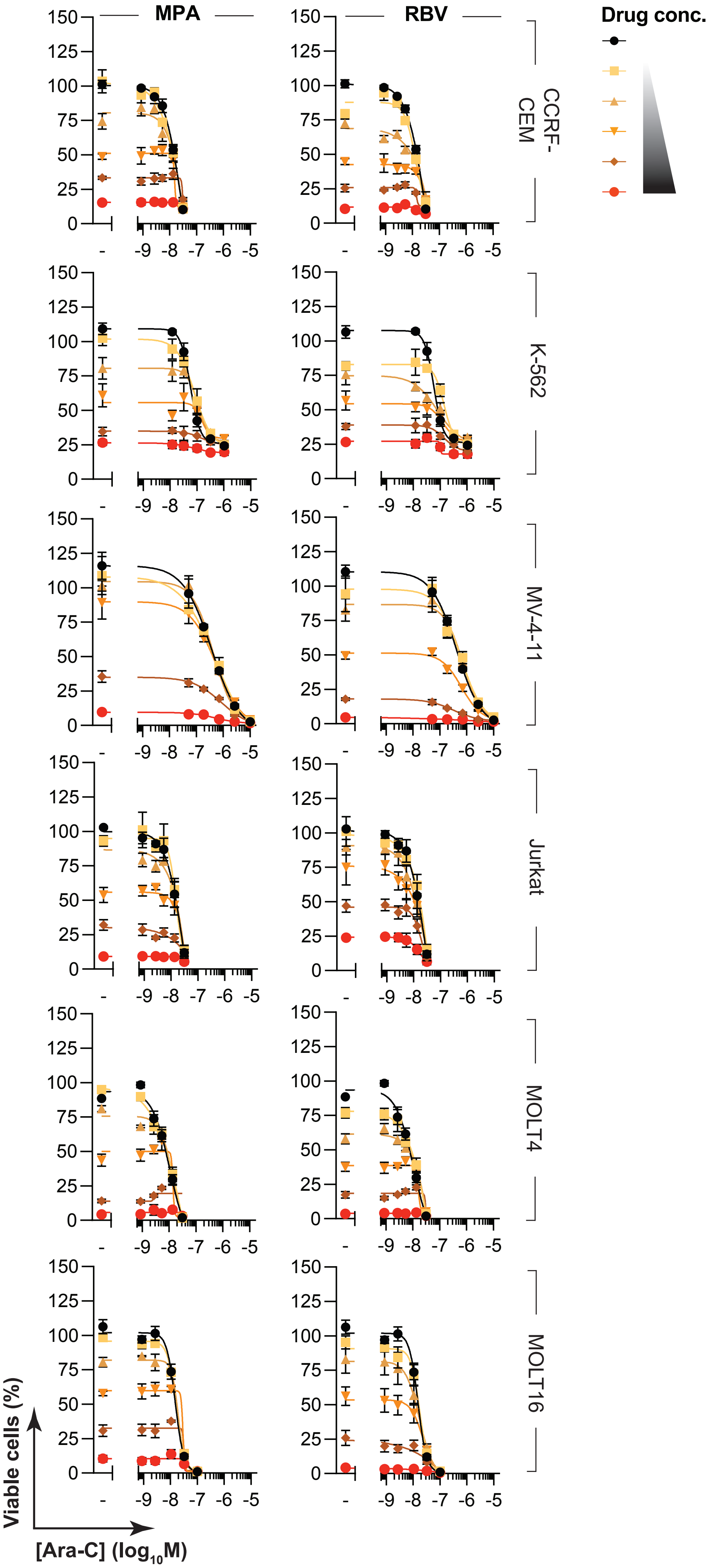

MPA
RBV
Drug conc.
CCRF-CEM
K-562
MV-4-11
Jurkat
MOLT4
MOLT16
Viable cells (%)
[Ara-C] (log_{10}M)

Figure S7. Dose-response curves from IMPDH inhibitor and cytarabine drug combination screen in a panel of haematological cell lines.

Cell viability measurements via resazurin reduction assay following 4-day treatment with cytarabine (ara-C) vs mycophenolic acid (MPA) or ribavirin (RBV) in a collection of 6 different haematological cancer cell lines, which includes CCRF-CEM, K-562, MV-4-11, Jurkat, MOLT-4 and MOLT-16. Drugs: CCRF-CEM – ara-C (0.001-0.03 μM), MPA (0.05-1 μM), RBV (1-100 μM); K-562 – ara-C (0.01-1 μM), MPA (0.05-1 μM), RBV (5-100 μM); MV-4-11 – ara-C (0.05-10 μM), MPA (0.05-1 μM), RBV (1-100 μM); Jurkat – ara-C (0.001-0.03 μM), MPA (0.05-1 μM), RBV (5-100 μM); MOLT-4 – ara-C (0.001-0.03 μM), MPA (0.05-0.5 μM), RBV (1-25 μM); MOLT-16 – ara-C (0.001-0.1 μM), MPA (0.02-2 μM), RBV (1-50 μM). Values represent mean ± SEM from 3 independent experiments performed in technical duplicates.

Figure S8

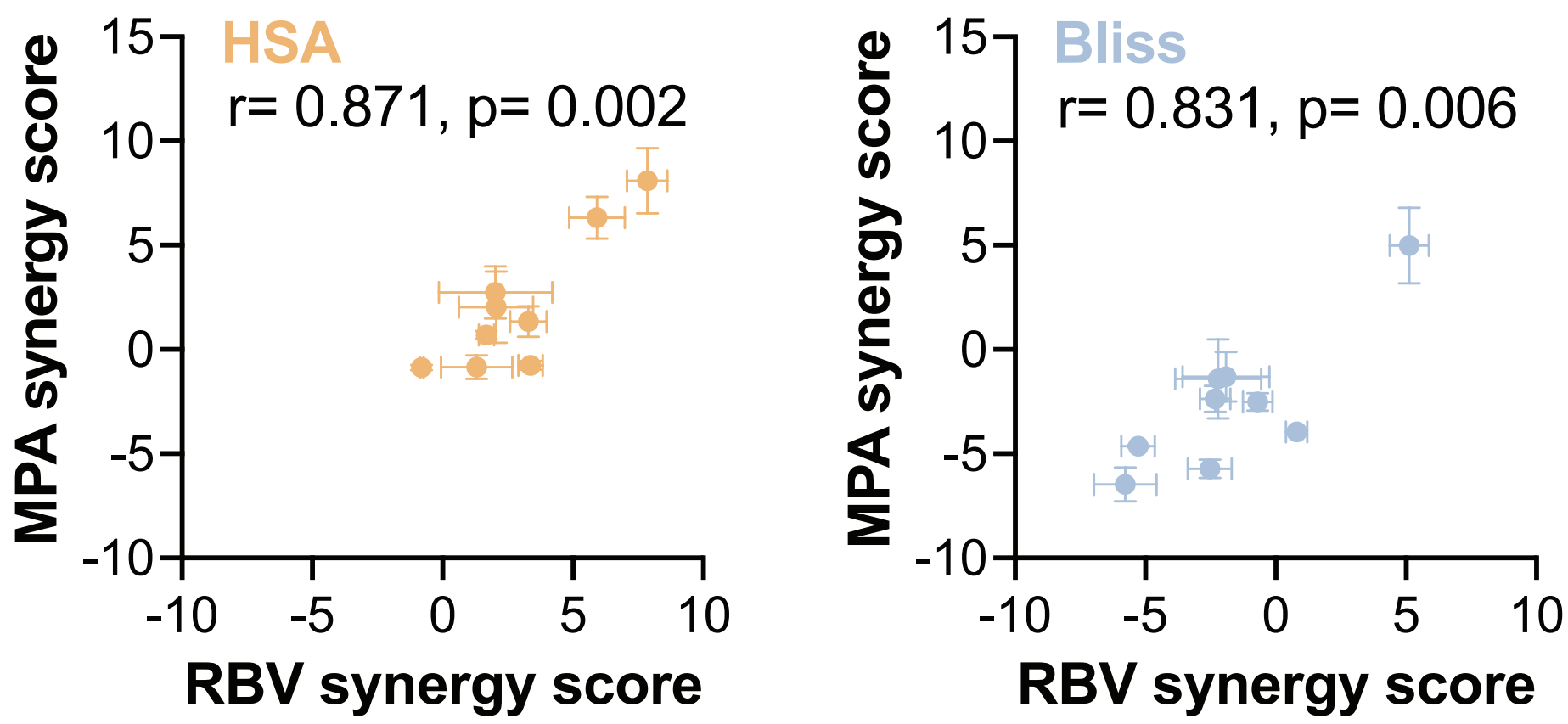

HSA
r= 0.871, p= 0.002
Bliss
r= 0.831, p= 0.006
MPA synergy score
RBV synergy score
15
10
5
0
-5
-10

Figure S8. Correlation of IMPDH inhibitor vs cytarabine synergy scores. Pearson correlation of synergy scores derived from different models (HSA, left; Bliss, right) obtained upon combined treatment of cytarabine (ara-C) and mycophenolic acid (MPA) vs synergy scores obtained from combined treatment of ara-C and ribavirin (RBV) performed in a collection of hematological cell lines (detailed in Figure 5). Each dot represents values (mean, n=3) derived from one cell line.

Figure S9

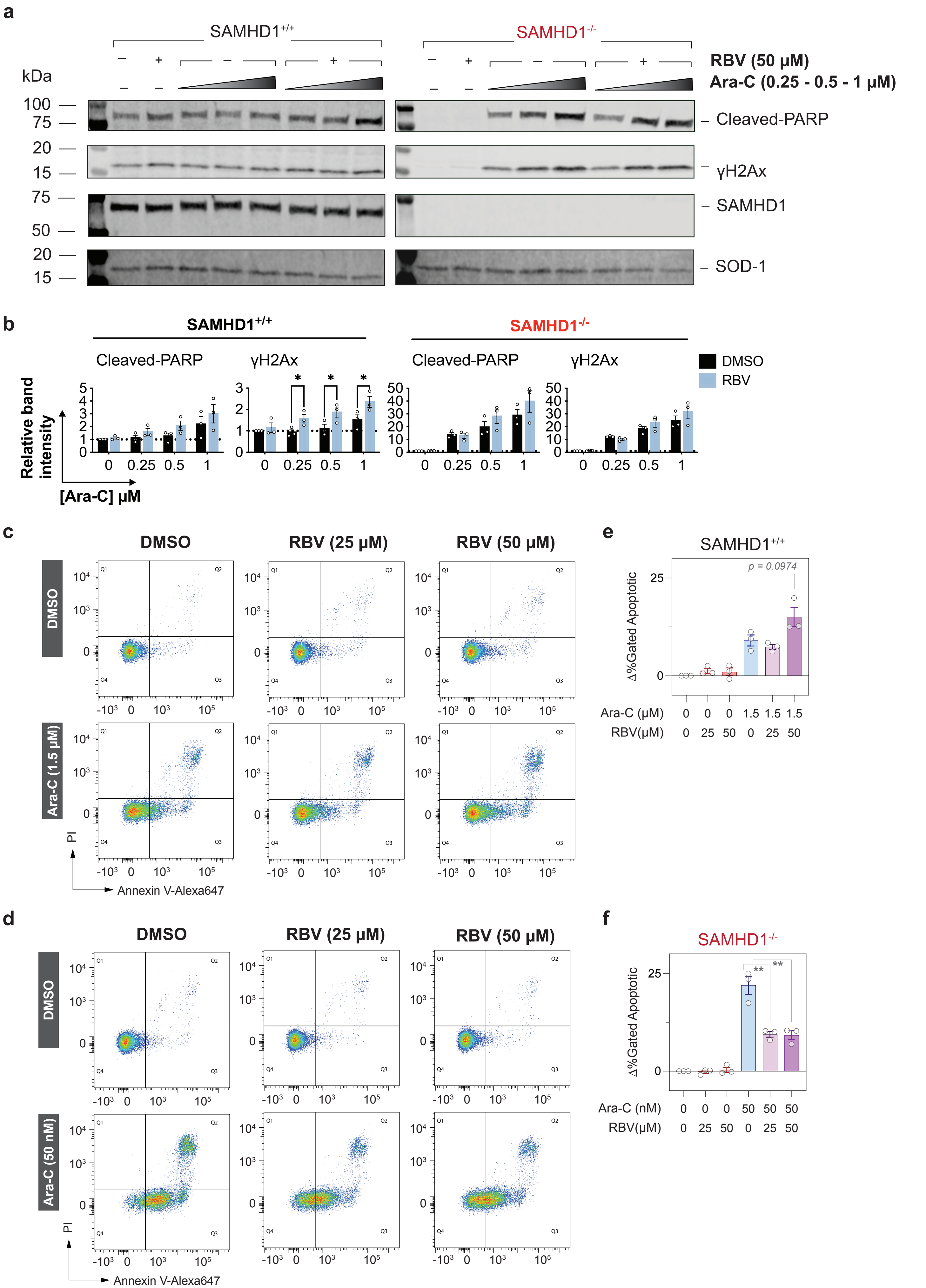

a
SAMHD1+/+
SAMHD1-/-
RBV (50 μM)
Ara-C (0.25 - 0.5 - 1 μM)
kDa
100
75
20
15
75
50
20
15
Cleaved-PARP
γH2Ax
SAMHD1
SOD-1
b
SAMHD1+/+
SAMHD1-/-
Cleaved-PARP
γH2Ax
DMSO
RBV
Relative band intensity
[Ara-C] μM
0 0.25 0.5 1
c
DMSO
RBV (25 μM)
RBV (50 μM)
Ara-C (1.5 μM)
PI
Annexin V-Alexa647
e
SAMHD1+/+
Δ%Gated Apoptotic
p = 0.0974
Ara-C (μM) 0 0 0 1.5 1.5 1.5
RBV(μM) 0 25 50 0 25 50
d
DMSO
RBV (25 μM)
RBV (50 μM)
Ara-C (50 nM)
PI
Annexin V-Alexa647
f
SAMHD1-/-
Δ%Gated Apoptotic
Ara-C (nM) 0 0 0 50 50 50
RBV(μM) 0 25 50 0 25 50

Figure S9. Activation of DNA damage and apoptotic signaling upon combined treatment with cytarabine and ribavirin.

(**a, b**) SAMHD1$^{+/+}$ or SAMHD1$^{-/-}$ THP-1 cells were treated 50 μM ribavirin (RBV) and different concentrations of ara-C (0.25, 0.5 and 1 μM) for 24 hours. Lysates were collected for immunoblot analysis with the indicated antibodies. Representative blot from three independent experiments shown in (a), mean band intensity relative to loading control plotted in (b), error bars indicate SEM. Two-tailed unpaired t-test, *$p<0.05$.

(**c-f**) SAMHD1$^{+/+}$ (c, e) or SAMHD1$^{-/-}$ (d, f) THP-1 cells were cultured with ara-C (1.5 or 0.05 μM, respectively) or DMSO, alone or in combination with RBV (25 or 50 μM) for 48 hours before apoptosis was assayed via Annexin V/PI staining followed by flow cytometry analysis. Representative flow cytometry plots shown (c, d), note DMSO controls are shared with experiment in Figure 6 as experiments were conducted together). Changes of Annexin-V/PI double-positive population compared to DMSO-only group (Δ%Gated) was determined, mean Δ%Gated ± SEM of n=3 independent experiments are plotted, together with values of individual experiments (e, f). Student's t tests (unpaired, two-tailed) were performed across treatment groups, specifically between MPA- and DMSO-treated groups, in the presence or absence of ara-C, where asterisk signifies statistical significance ($*p \leq 0.05$, $**p \leq 0.01$).

Figure S10

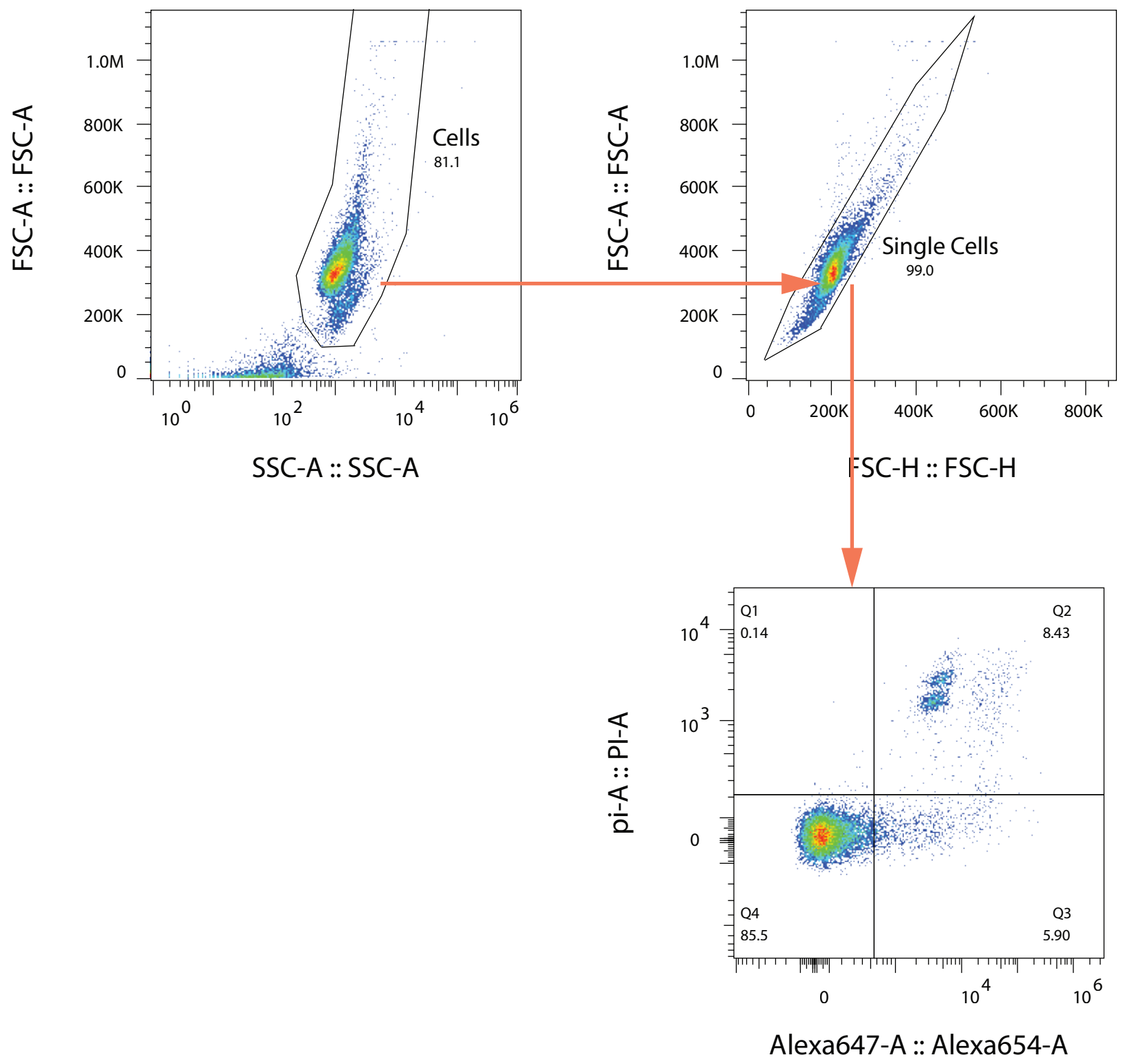

Cells
81.1
Single Cells
99.0
Q1
0.14
Q2
8.43
Q4
85.5
Q3
5.90
FSC-A :: FSC-A
SSC-A :: SSC-A
FSC-H :: FSC-H
pi-A :: PI-A
Alexa647-A :: Alexa654-A

Figure S10. Flow cytometry gating strategy

Cells were gated out based on forward (FSC) and side (SCC) scattering, followed by doublet discrimination based on the FSC height (FSC-H) and area (FSC-A). Viable (Q4), early apoptotic (Q3), apoptotic (Q2), and necrotic cells (Q1) were then identified via quadrant gating on the single cell population, based on Annexin-V-Alexa 647 and PI signals.

Figure S11

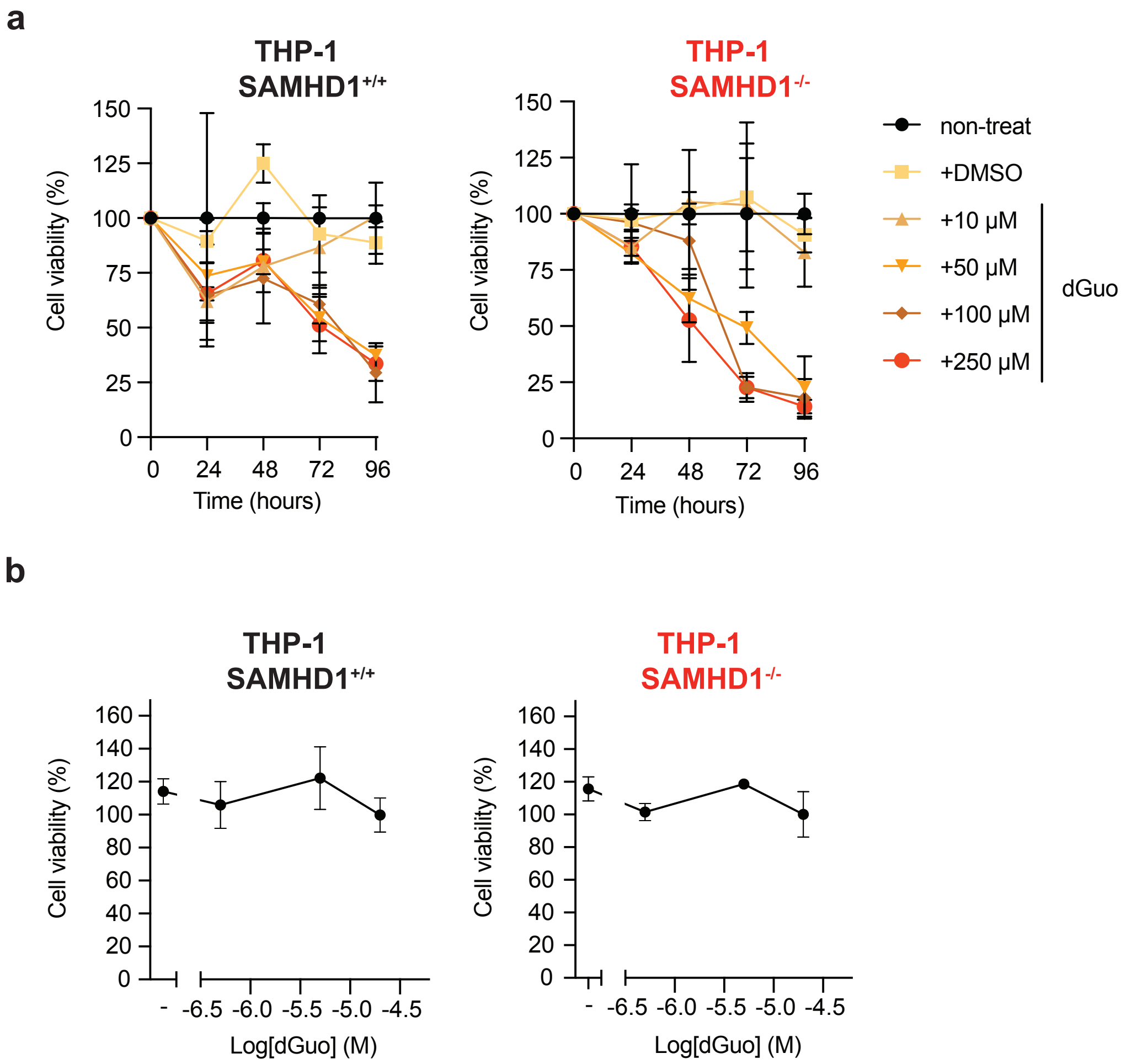
a
THP-1 SAMHD1+/+
THP-1 SAMHD1-/-
Cell viability (%)
Time (hours)
non-treat
+DMSO
+10 µM
+50 µM
+100 µM
+250 µM
dGuo
b
THP-1 SAMHD1+/+
THP-1 SAMHD1-/-
Cell viability (%)
Log[dGuo] (M)

Figure S11. Titration of deoxyguanosine and cell viability measurements.
(**a**) SAMHD1 proficient (SAMHD1$^{+/+}$) and deficient (SAMHD1$^{-/-}$) THP-1 cells were cultured for 96 hours with increasing concentrations of deoxyguanosine (dGuo; 10, 50, 100 and 250 μM). Every 24 hours, viable cells were counted using Trypan Blue solution and percentage of viability was then calculated using the untreated cells as positive control (100%). Graph shows mean values from 2 independent replicates and error bars indicate SEM.
(**b**) Effects of different concentrations of dGuo (0.5, 5 and 20 μM) were assessed on THP-1 SAMHD1$^{+/+}$ and SAMHD1$^{-/-}$ cells in a total of three independent experiments, mean values $\pm$ SEM plotted. Cells were treated for 96 hours and viability measured using the resazurin reduction assay.

Figure S12

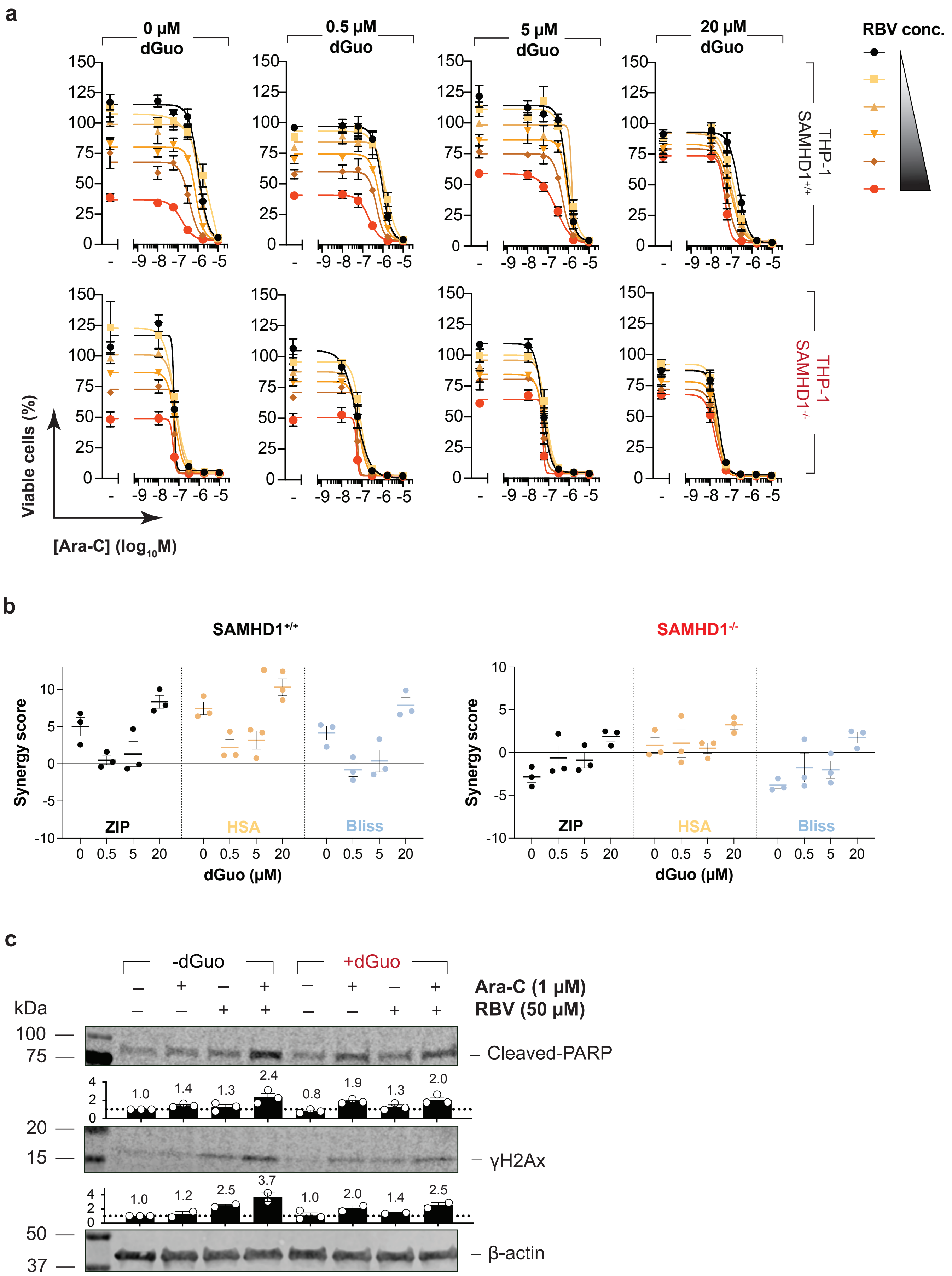
a
0 μM dGuo
0.5 μM dGuo
5 μM dGuo
20 μM dGuo
RBV conc.
THP-1 SAMHD1+/+
THP-1 SAMHD1-/-
Viable cells (%)
[Ara-C] (log10M)
b
SAMHD1+/+
SAMHD1-/-
Synergy score
ZIP
HSA
Bliss
dGuo (μM)
c
-dGuo
+dGuo
Ara-C (1 μM)
RBV (50 μM)
kDa
Cleaved-PARP
1.0
1.4
1.3
2.4
0.8
1.9
1.3
2.0
γH2Ax
1.0
1.2
2.5
3.7
1.0
2.0
1.4
2.5
β-actin

Figure S11. Effect of deoxyguanosine supplementation upon ribavirin and cytarabine combination treatment.

(**a**) Proliferation inhibition analysis of cytarabine (ara-C, 0.01-10 μM) and ribavirin (RBV, 0.5-5 μM) treated SAMHD1$^{+/+}$ and SAMHD1$^{-/-}$ THP-1 cells supplemented with the indicated concentrations of deoxyguanosine (dGuo). Mean values plotted ± SEM from 3 independent experiments each performed in duplicate.

(**b**) Drug synergy plots for ara-C and RBV derived from treatments in (a). Each data point indicates an average delta score from the indicated synergy models from a single dose–response matrix experiment performed in duplicate. N=3, mean ± SEM represented with horizontal and vertical bars, respectively.

(**c**) SAMHD1$^{+/+}$ THP-1 cells were cultured for 24 hours with the indicated doses of ara-C and/or RBV with or without dGuo (5 μM) before harvesting for immunoblot analysis with the indicated antibodies. Representative cropped immunoblot from three independent experiments shown. Mean band intensity (n=2-3) relative to loading control and normalised to untreated shown below.
